# Identifying Past Beer Production: Contributions from an Ethnoarchaeological Study in Bedik Villages, Senegal

**DOI:** 10.1080/19442890.2024.2334509

**Published:** 2024-04-16

**Authors:** Pauline Debels, Julien Vieugué, Thomas Pelmoine, Moustapha Sall, Anne Mayor

**Affiliations:** a Faculty of Sciences, Laboratory ARCAN (Archaeology of Africa & Anthropology), University of Geneva, Geneva, Switzerland; b CNRS - UMR 8068 TEMPS (Technologie et Ethnologie des Mondes Préhistoriques), MSH Mondes, Nanterre, France; c Inrap NA&OM, Poitiers, France; d Faculty of Letters and Human Sciences, Department of History, University Cheikh Anta Diop, Dakar, Senegal; e University of Geneva, Global Studies Institute (GSI), Geneva, Switzerland

**Keywords:** Sorghum beer, architecture, pottery, morphometry, use-alteration, Senegal, historical approach, cross-cultural analogies

## Abstract

The identification of beer production in past societies remains a challenge as very few studies have discussed its material evidence. Our investigation in Senegal aimed at filling this gap. We documented 14 beer houses and several beer cooking areas in five Bedik villages and excavated a beer house and associated cooking area in a recently abandoned village. We recorded the architectural attributes of the beer-making structures (location, shape, size, materials, techniques, internal layouts). We also analyzed associated pottery combining typometry and use-wear. Such an integrated study revealed that the pottery types (large vessels, small bottles) and use-alteration (inner non-abrasive attrition), are the most distinctive features for identifying beer production, besides the beer houses’ internal layouts (wedge holes of large pottery, altar) and the beer cooking areas’ location outside the compound. Exploration of the same criteria in other cultural contexts in Africa lends support to the broader significance of these findings.

## Introduction

Beer consumption is very important in African non-Muslim societies, where it is used as a token of hospitality, as reciprocation for voluntary work, and in collective and religious celebrations linked to the annual cycle of initiations and cultivation (Arthur 2003; Berger 2022; Jolly 2004). The production of this high-calorie beverage is time-consuming and needs a significant fraction of cereal harvests. It involves a diversity of recipes and ingredients, including cereals like millet, sorghum, and maize in the savannas, and various tubers or legumes in the tropical forests. It takes place in specific locations, lasts several days, and involves various containers, such as ceramic vessels used for cooking, fermenting, transporting, and serving the beverage.

The identification of beer production in archaeology is therefore key to understanding past social, economic, and religious organization and holds significant interest surpassing cultural contexts (Arthur 2022). In the Nile Valley, the importance of beer is attested from the Predynastic period in the fourth millennium BCE (Farag et al. 2019), based on textual and archaeological evidence, as well as organic residue analysis (Farag et al. 2019; Heiss et al. 2020; Michel, McGovern, and Badler 1993; Perruchini et al. 2018; Wang, Friedman, and Baba 2021). However, evidence of ancient beer production in Sub-Saharan Africa beyond the Nile Valley remains scarce due to various challenges. First, it is difficult to identify in the archaeological record. Some archaeologists have suggested an increase of beer production from the mid-first millennium CE in the Diamaré (Cameroon) by drawing a parallel between the shape of archaeological pottery and present-day ceramic vessels linked to beer production (Langlois 2005). Similar comparisons have been made to prove the production of beer in Kirikongo (Burkina Faso) during the period dated 100 to1650 CE (Dueppen and Gallagher 2021). Second, written sources are rare and recent in Sub-Saharan Africa compared to the Nile Valley. A few Arabic sources from the tenth century CE mention beer consumption among the Zaghawa in Chad and in the Ghana and Mali kingdoms (Cuoq 1985). Later, information can be found in European accounts by travelers or missionaries since the seventeenth century CE, and in studies made by social anthropologists among various African societies during the twentieth century (Berger 2022). Unlike the Nile Valley, no residue analysis has been conducted so far on Sub-Saharan archaeological pottery assemblages in search for fermentation biomarkers.

Despite the huge importance of beer in numerous societies, few ethnoarchaeological studies (Arthur 2002, 2003) have focused on studying beer materiality and spatiality. This research is nonetheless critical given the rapid disappearance of traditional beer production techniques using local materials like pottery, basketry, and calabashes, the progression of Islam, the availability of industrial beers, and the use of imported metal or plastic containers, alongside the loss of pottery know how. We are thus facing a lack of systematic ethnoarchaeological references linking material culture and beer production, and the urgency to build them. Ethnographic investigations are crucial to help establish diagnostic criteria for the interpretation of beer production in archaeology, even if post-depositional mechanisms and local developments may complicate the establishment of analogies across important spatial, temporal, or cultural distances. Projects endorsing a methodology combining both ethnographic investigations and the excavation of recent contexts (such as Arthur 2021) have the potential to identify the archaeological expression of beer production, to test the preservation of material culture, and to highlight past know-how, as well as social and ritual aspects.

In this manner, our ethnoarchaeological study aims to explore the question of the identification of beer production in African past societies, looking at its spatiality and materiality. Our main questions are the following: Which are the locations, the structures, and the material cultures associated with the different stages of the beer manufacturing process? What sets beer houses and hearths apart from the other types of buildings and fireplaces in terms of architecture and pottery? What are the most diagnostic criteria for identifying beer production in past societies?

To answer these questions, an ethnoarchaeological study was conducted in several Bedik villages located in south-eastern Senegal. This region deeply integrates beer production into societal norms, shaping the calendar and even featuring dedicated structures to house this practice. An ethnographic study first mapped the location of the beer production stages, documented the architecture of beer houses and hearths, and analyzed the pottery assemblages related to these specialized structures. An archaeological excavation was then conducted on the site of Eguong, a Bedik village recently abandoned. The collapsed beer house and hearth as well as the fragmented pottery assemblage from the site were documented following the same approach. Given the temporal and spatial proximity of these contexts, direct analogies could be made. The diagnostic traits for identifying beer production inside Bedik villages were finally compared with those from other beer-making societies in West, East and South Africa to test their cross-cultural validity.

## Bedik Society and Beer

### Geographical and Cultural Contexts

**Figure 1.**
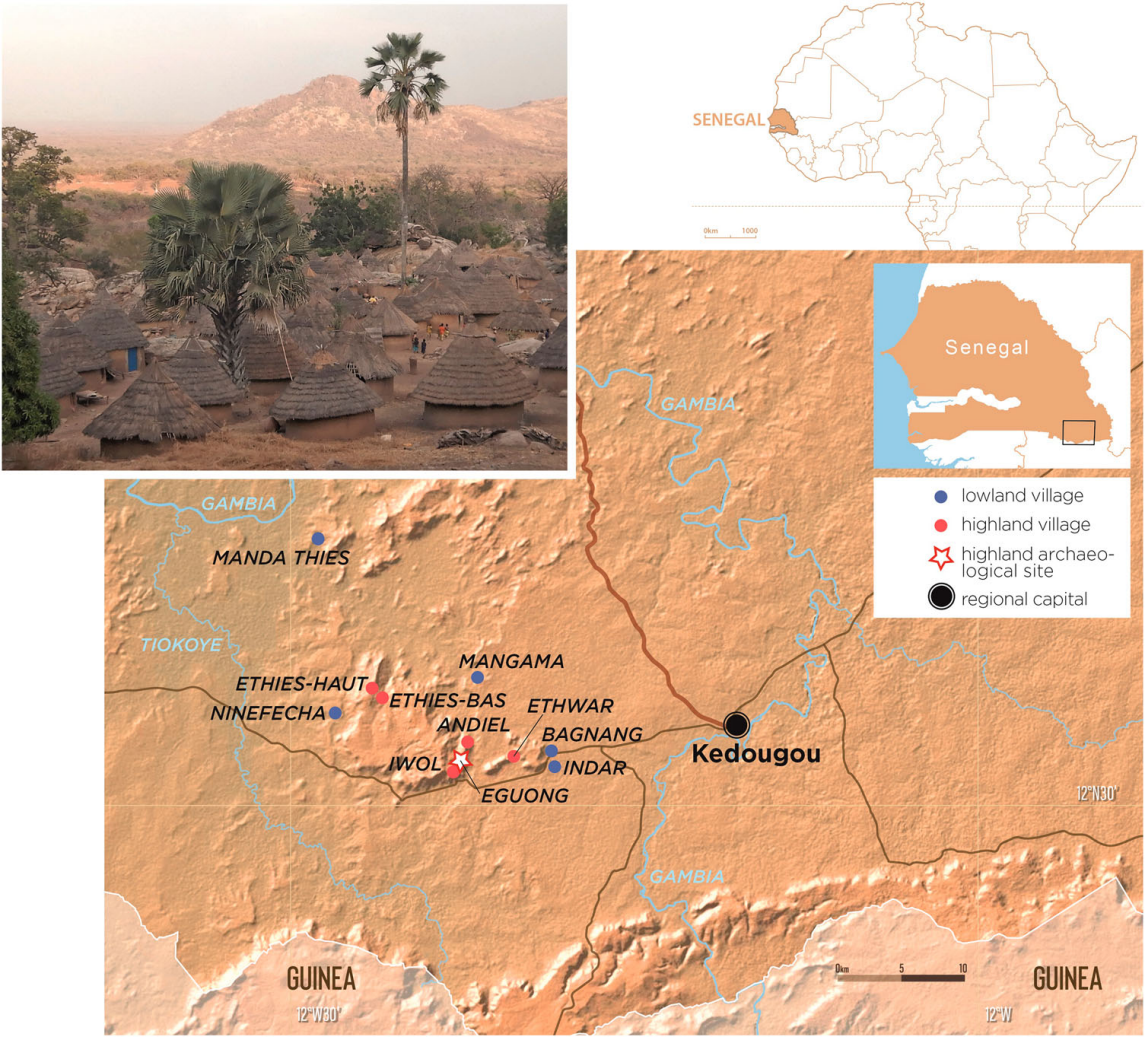
Location of the main Bedik villages studied in south-east Senegal.

The Bedik country lies in south-eastern Senegal, between the regional capital of Kedougou to the west, the border with Guinea to the south and the Niokolo Koba Park to the north. It is bounded by the Gambia River and one of its tributaries, the Tiokoye, and by the last foothills of the Fouta Djallon Mountain (Figure 1). It forms part of a wider area designated as a UNESCO World Heritage Site in 2012 under the title “Bassari Cultural Landscapes”, including territories also inhabited by the Bassari, Fulani, and Djalonke people (Bocoum and Moriset 2012). The territory inhabited by the Bedik is around 300 km^2^, located in the administrative *arrondissement* of Bandafassi.

The territory occupied by the Tenda populations (including the Bassari and Bedik people) was previously much larger than today, but following the insecurity engendered by the slave raids of the Islamized Fulani of Fouta Djallon in the nineteenth and early twentieth centuries, the Bedik settled in refuge villages perched on rocky reliefs (Ethwar, Andiel, Iwol, and Ethiès), connected to caves used as hiding places in case of attacks. As the twentieth century progressed and the region was pacified, the temporary farming hamlets on the plains became permanent villages (Ferry 1967). While some highland villages are still well populated, others are completely deserted, in favor of the lowland seasonal hamlets, which became sedentary villages. They are now frequented only for the ceremonies and initiations that punctuate the Bedik annual calendar.

The ethnogenesis of the Bedik has developed over the last few centuries through migrations from the Mali empire, beginning in the thirteenth century, and the aggregation of Keita and Kamara clans, including Samura, Sadiaxu, and Kante families, each having a particular power or knowledge. Thus, the Keita are the political chiefs, the Sadiaxu the religious chiefs, the Kamara play the role of their adjuncts, assistants or intermediate organizing ceremonies, and the Samura and the Kante are the blacksmiths (Gomila and Ferry 1966). However, unlike neighboring societies, Bedik society is not structured into endogamous castes. Matrimonial alliances and craft activities are therefore freely chosen. Nearly all inhabitants claim to be Christians but remain intimately linked to the ancestral religion. They speak the Menik language, divided into two main dialects, Biwol and Banapas.

Bedik people traditionally eat a variety of dishes daily based on domestic cereals (such as sorghum, fonio, maize, and rice) and tubers (like yams and groundnuts), often served with a sauce made mainly from wild plants (such as baobab leaves or complex preparations of different grains) and hunted meat (warthogs, monkeys, antelopes, etc.). Domestic animals (chickens, goats, and cows) are used only for sacrifices and collective feasts. Bedik mainly drink water and palm wine on a regular basis, and beer only for ceremonies. Recipes, ingredients, and contexts of consumption have been rapidly changing for one or two decades (Olowodun et al. 2024).

### Context of Beer Production and Consumption

#### Socio-Economic and Symbolic Context of Beer Consumption

In the Bedik country, beer (*ungotyin)* is not a secular fermented beverage drunk daily, as it is the case of palm wine. It is only consumed during the dry season for ceremonies and during the rainy season for collective work in the fields, sometimes accompanied by masks. Beer is produced six or seven times a year for feasts linked to the agricultural calendar: when the millet begins to ripen (November), when it is possible to burn the grass around the villages (December), and at the end of the harvest (end of December), as well as for feasts linked to the circumcision (March), the initiation of young circumcised men (April) and the women's festival (May). In addition, each family makes beer for at least two collective works, one to cultivate the woman's fields and another one to cultivate the man's fields. The frequency of beer production can, therefore, be estimated at around ten times a year.

Beyond its obvious symbolic dimension, beer production has a huge impact on the socio-economic system of the Bedik society. In March 2022, ethnographic survey was conducted in the village of Iwol (618 inhabitants in 2022) while beer was being prepared for the circumcision feast. Interviews carried out with the inhabitants have allowed us to estimate the ratio between sorghum for beer and sorghum for food per year. To produce beer for the six annual feasts and a few collective works in the fields, a medium-size family uses from 265 to 465 kg of sorghum, depending on whether it has a young, circumcised boy to initiate. In addition to other cereals and tubers (500 kg of maize, 200 kg of fonio, 1000 kg of groundnuts, and 50–100 kg of rice, depending on means), the family needs 500 kg of sorghum a year for food. These data tend to show that almost half a family's sorghum harvest can be spent to make beer. Beer mobilizes, therefore, an important part of the sorghum production. If needed, maize can complement sorghum for beer production.

Observations and interviews also have allowed us to assess the volume of beer produced for the circumcision feast organized in March 2022. Eight Keita, Kamara, and Samura families, comprising one to three circumcised boys aged 14–16, cooked between 800 and 2400 l of malt, and at least three families without circumcised boys prepared a 200-l barrel each on a voluntary basis to help. Two sessions of beer were made a day apart to have enough to drink for the whole three to four-day feast, bearing in mind that consumption must take place preferably within one to two days after fermentation and maximum of three days. Thus, at least 9245 l of malt were boiled, mostly in 200-l metal barrels, but can be estimated to amount to a total of 10,000 l, knowing few barrels were likely not observed. At the end of the beer-making process, we have measured 400 l of malt, resulting in 170 l of ready-to-drink beer, i.e. a reduction of more than half in volume. A quick calculation shows that around 4250 l of beer were served in Iwol to the inhabitants of the village (men, women, and children), as well as to guests coming from other Bedik villages for this feast, which means an enormous economic effort for enhancing social cohesion.

#### Spatiality and Materiality of Beer Production.

**Figure 2.**
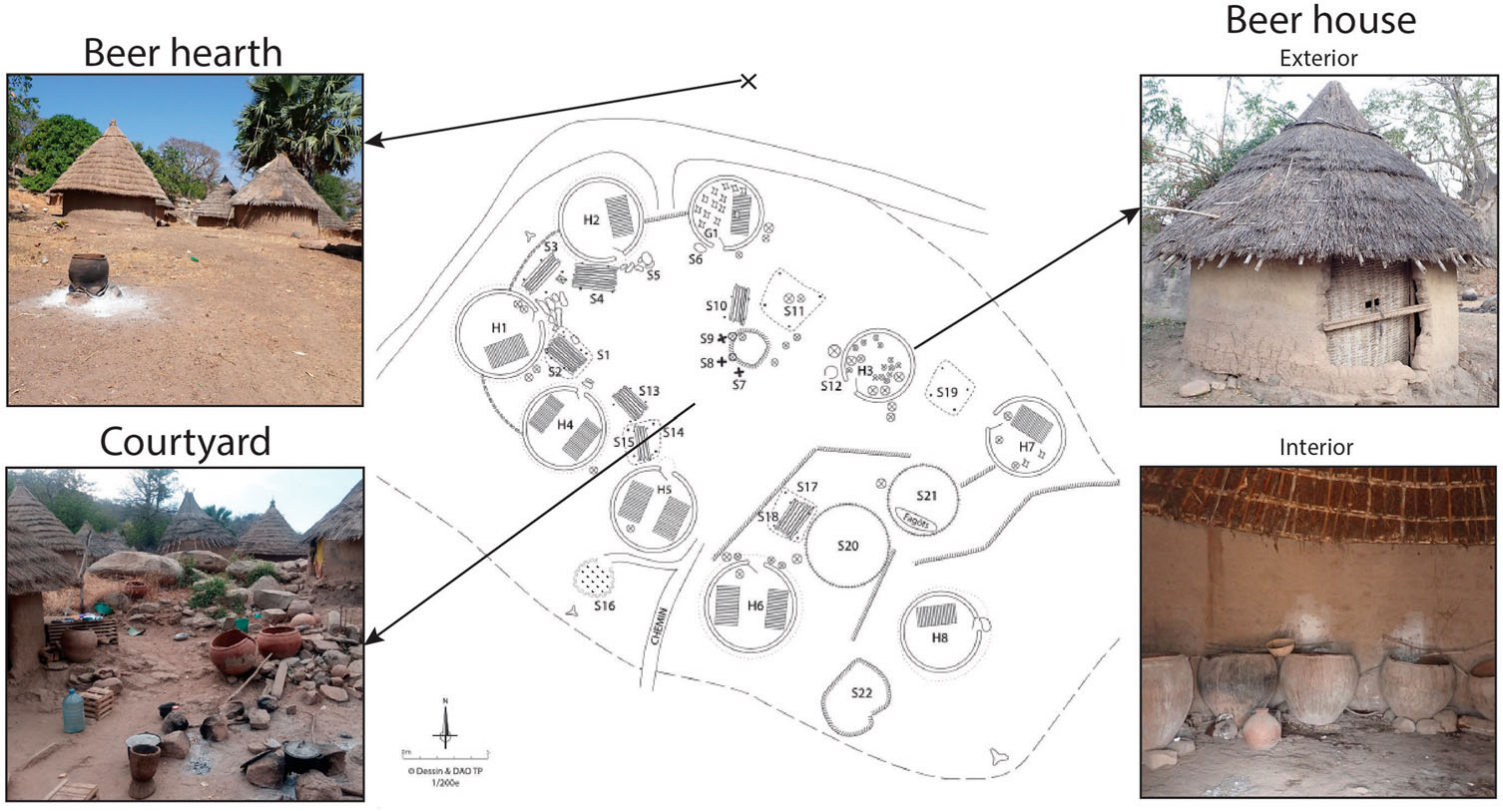
Location of the different places of beer production inside a Bedik compound (photographs come from different compounds).

Beer-making takes place in three different areas of the Bedik compounds (Figure 2).

**Figure 3.**
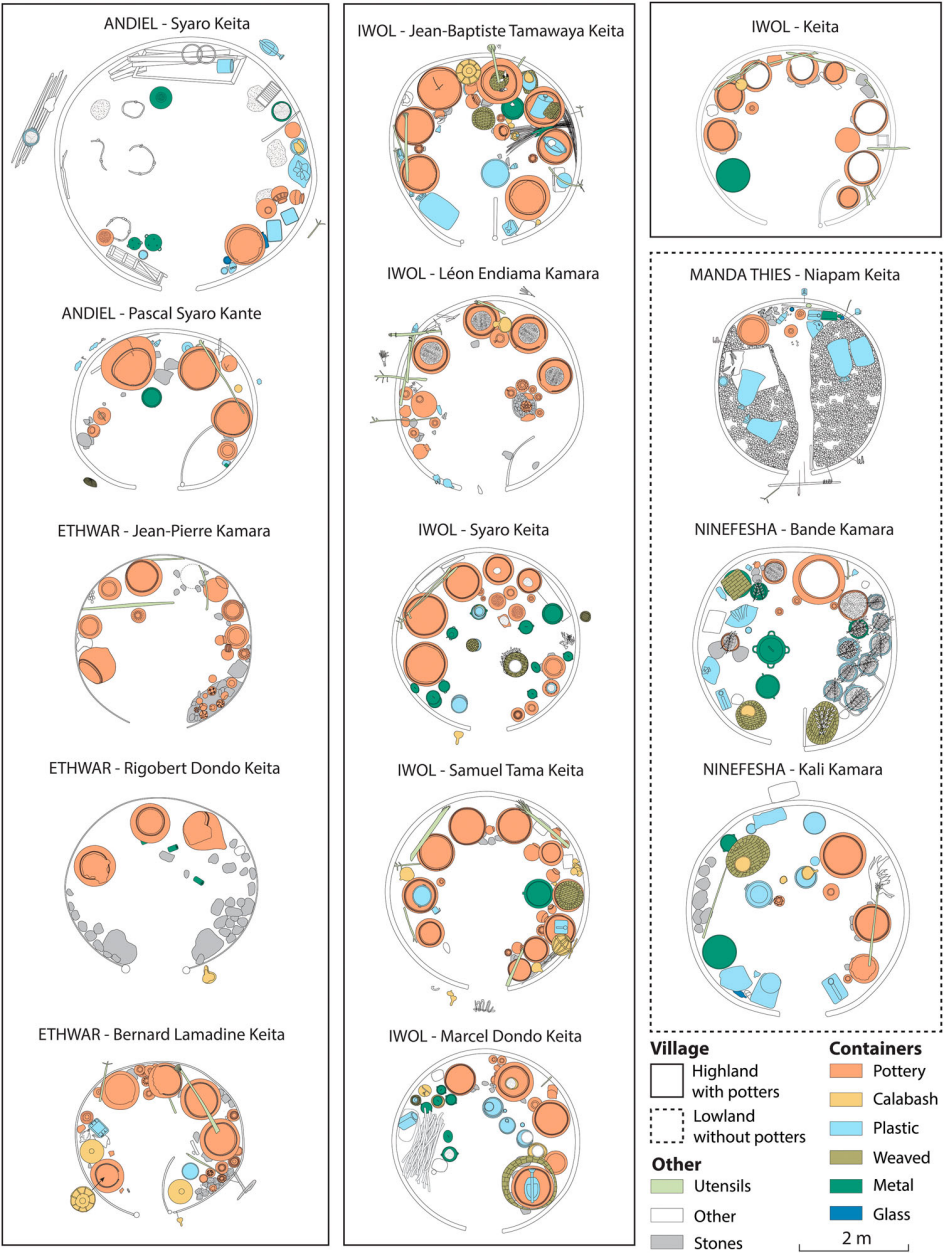
Plans of the 14 beer houses documented in the Bedik country. The spatial distribution of beer pots and utensils inside these buildings is detailed.

1. The open-air courtyards are located at the center of the compound. They usually accommodate a variety of food activities (including preparing, cooking, and eating meals as well as making beer) and handcraft activities (such as manufacturing pottery or basketry). No structure linked to beer production is arranged in this space.
2. The beer houses are situated among the other circular buildings. They are usually part of a compound inhabited by an extended family to which the different utensils used for beer-making belong. If a Bedik family does not own a beer house, they may give their sorghum to neighbors and the resulting brew is shared. Beer houses contain various calabashes and basketry, pottery vessels, wooden sticks, metal, and plastic containers related to beer production (Figure 3; Supplement 1). Large ceramic vessels used for malting, brewing, and fermenting beer are distributed alongside the wall of the beer houses, like the small pottery used for fermenting and transporting. Although large pottery vessels are mostly static due to their size and weight, they are occasionally moved to different places, are lent or taken out for refurbishing. Small beer pots are highly mobile as they are particularly used for transporting beer to the consumption place, inside or outside the compound, in the village. Some beer houses hold an altar inside, composed of special stones and specific pots. Very few beer houses, only used for specific celebrations, are without any pottery inside. The village of Iwol holds, for example, an empty beer house dedicated to women and another to men, used respectively during women's and men's feasts, which do not occur every year. For these special occasions, beer vessels are borrowed from Kamara families as they are responsible for rituals. The material culture found inside beer houses is, therefore, overwhelmingly associated with beer production. In some exceptional cases only, the beer houses can be used as granaries or as an opportunistic brooding area for hens. They may contain objects that are not related to beer-making.
3. The beer hearths are on the outskirts of the compound, a few meters apart. These cooking areas are made up of simple structures composed of three large stones with no flooring or superstructure. Large vessels used to cook malt are usually either found on top of the stones or close to the hearth, upside down on their rim. The large hearths involved in the beer-making can occasionally be used to prepare soap in a big vessel used specifically for this purpose, as soap requires long cooking sessions.

#### Beer-Making among the Bedik

The manufacturing process of beer was documented in the village of Iwol in March 2022. Observations were completed in Indar in July 2023.

##### Ingredients and Utensils

Beer-making involves a large quantity of cereals and water. According to our ethnographic observations, the production of 340 l of this fermented beverage requires 200 kg of sorghum and 800 l of water. Beer-making also requires the use of a wide variety of kitchen utensils and containers, including:

- Ceramic vessels that can be divided into three main types: (1) slightly closed shapes of medium to large size (65–150 l) with a pointy base used for cooking malt (*atieda* in Biwol*, gatieda* in Banapas); (2) slightly closed shapes with a large to very large capacity (between 65 and 360 l) with a pointy base used for malting, brewing and fermenting beer. They come in two main sizes that are called differently accordingly (*elema*, large; *nianema*, small). Rare smaller ones can also be found (called *nietede*, not documented in this study). *Elema* is often used as a generic term for the category; (3)very closed shapes of small to medium size (1–20 l) with a round base used for fermenting, transporting, and serving beer. They come in two main different sizes that are called accordingly (*niene*, small; *amband* in Biwol, *gamband* in Banapas, medium*).* Rare larger ones with a volume of 20–25 l can also be found (called *ide*, not documented in this study). *Niene* is often used as a generic term for the category.
- Utensils made from organic materials such as calabash bowls and ladles, wooden mortars, and pestles as well as sticks, mats, basketry, and specific filters made from palm leaves and split bamboo stems.
- Containers made from imported materials, such as metal barrels and plastic basins and cups.

The introduction of metal and plastic containers is recent. These objects tend to supplement some calabash or pottery containers and sometimes replace them entirely depending on the function. For instance, beer is traditionally cooked inside large ceramic vessels (*atieda*); today, this functional type is frequently replaced with metal barrels which have a better heating efficiency, reducing the cooking time and the amount of combustible material, while being more resistant to thermal and mechanical shocks. Similarly, beer is traditionally consumed in calabash bowls but today, these containers tend to be supplemented but not totally replaced with plastic bottles and cups.

Contrarily, some functional types of pottery, like the large conical vessels (*elema*) and the small globular vessels (*niene*) used for fermentation, cannot be replaced by containers made from other materials, because of the performance of the clay material for trapping yeast in the porous walls. The large beer pots are known to be difficult to fashion, and only a few old potters still have the skills to produce such jars, but they are never replaced with metal barrels, because these containers appear to be unsuitable for fermentation. *Elema* are replaced either by new ones or sometimes by old large *atieda* initially used to cook the wort.

##### Preparation Time and Procedure

The beer-making process among the Bedik people is intricate and spans approximately 10 days. It involves three main stages: malting, brewing, and fermentation (Figure 4). Figure 4.Manufacturing process of beer among the Bedik: (a) Malting, (b) Brewing, (c) Fermentation. Pottery vessels are colored while organic, plastic and metal containers are shown in outlines.

**Figure:**
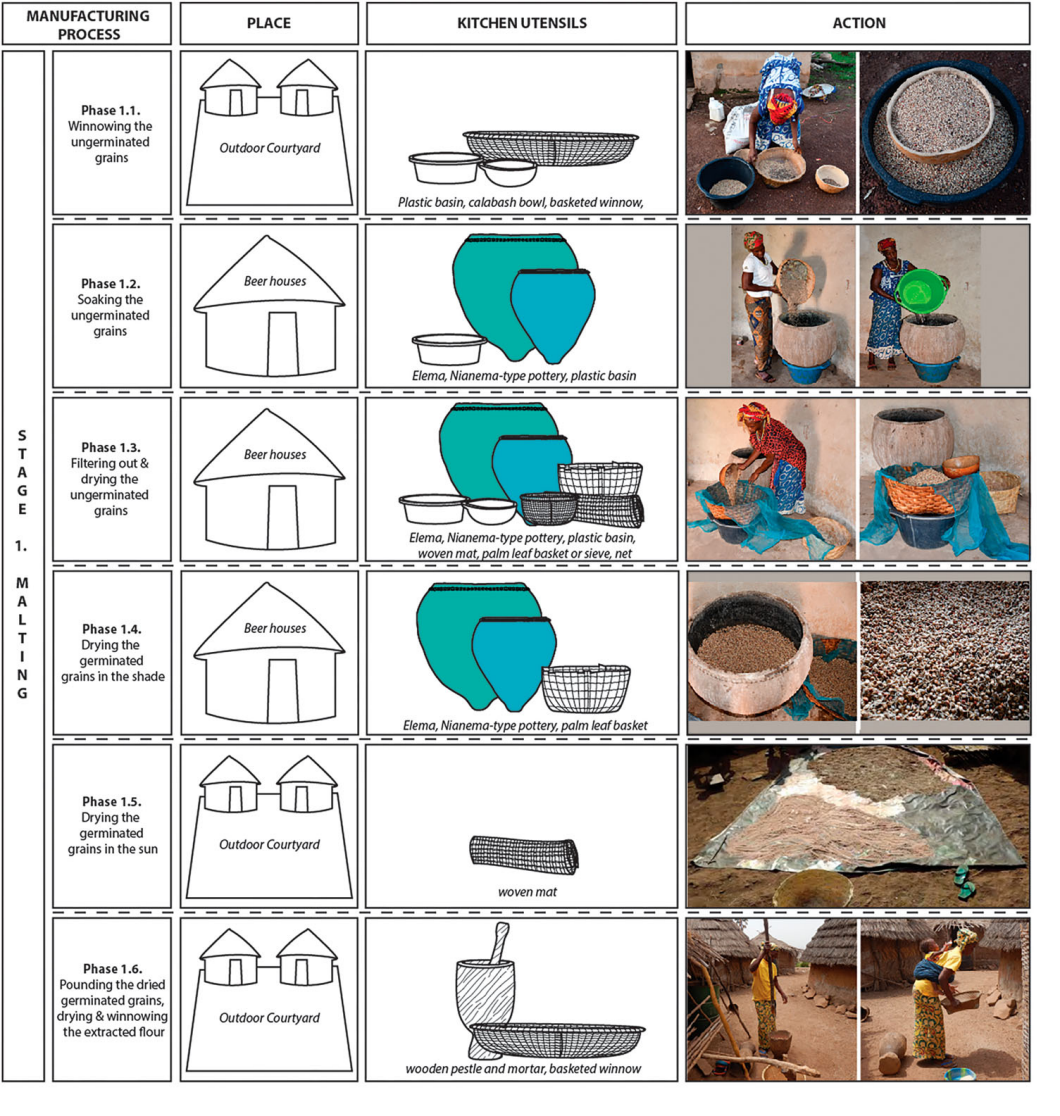

**Figure:**
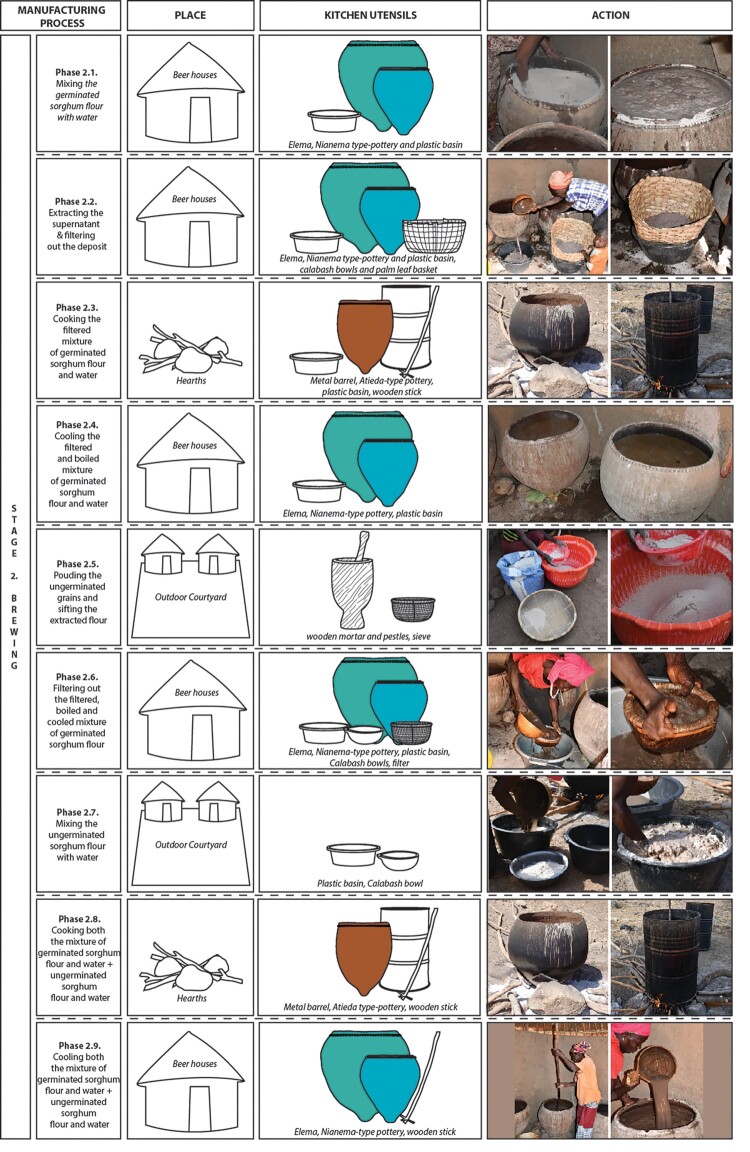

**Figure:**
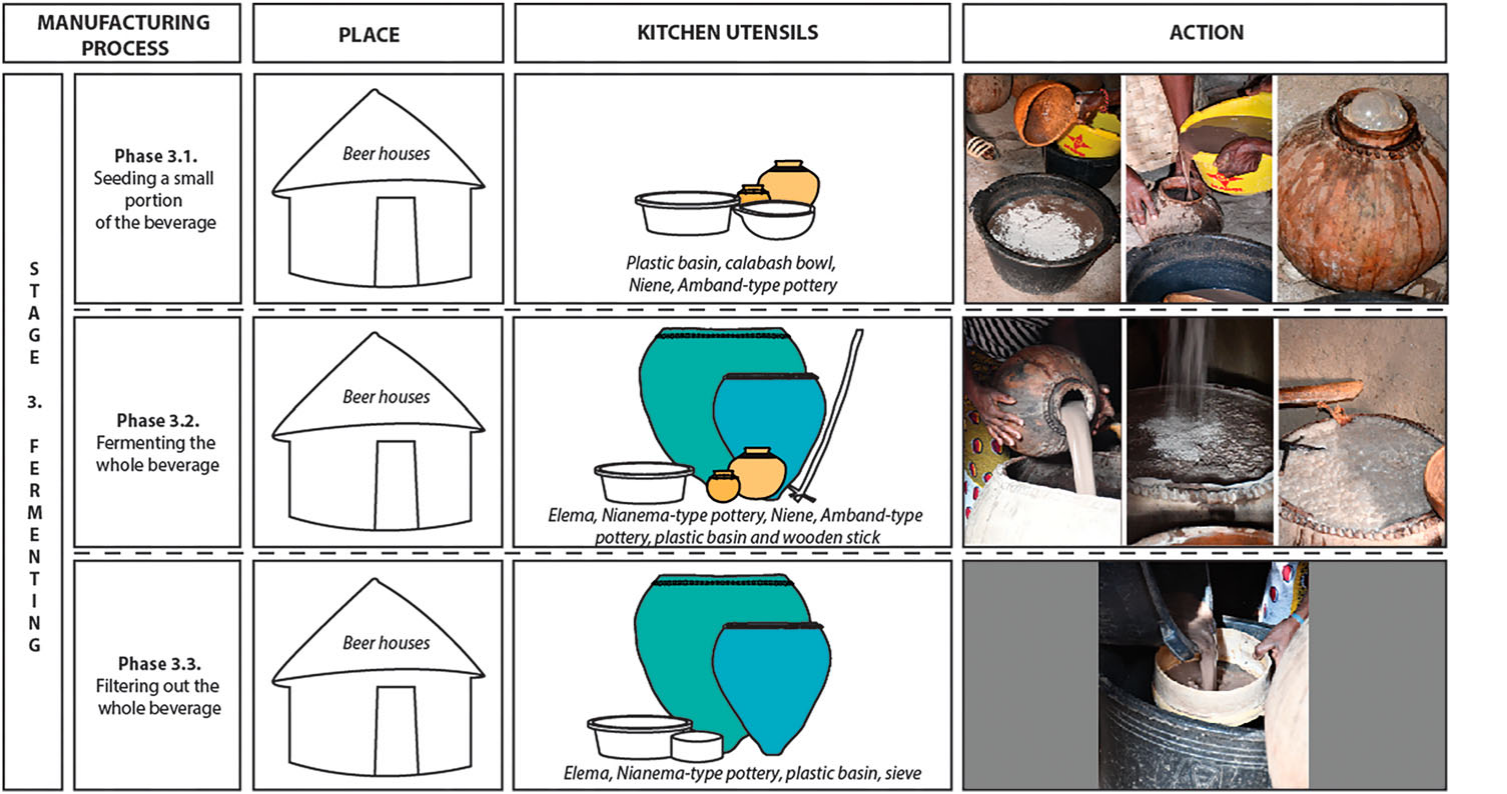

**Stage 1. The malting (6 days)** consists of preparing the grains for the chemical reactions that will enable the starch contained in the cereals to be saccharified (Jolly 2004). It is carried out in six main phases (Figure 4a): **1.1.** 150 kg of sorghum grains are winnowed in the courtyard to remove the small part of grains unsuitable for beer-making. **1.2.** The winnowed sorghum grains are soaked for 1 d in *elema* pots inside the beer house. **1.3.** The soaked and winnowed sorghum grains are filtered using a fine mesh net placed above a palm-tree leaf basket. They are then distributed among several *elema* and baskets inside the beer house and left to dry for 2–3 days. The grains start to germinate. **1.4.** The germinated sorghum grains are put all together in the *elema* and left to dry for one extra day inside the beer house. **1.5.** The germinated sorghum grains, so-called green malt, are spread out on a mat in the courtyard and left to dry in the sun for 1–2 days. **1.6.** The dried germinated sorghum grains are pounded using wooden mortars and pestles in the courtyard. The flour obtained is then spread out on a mat and left to dry for 3–4 h. Once dried, it is winnowed to remove the coarse particles (fragments of partially crushed grains, etc.).

**Stage 2. The brewing (3 days)** aims at obtaining a fermentable wort after solubilizing the malt flour and transforming the starch (Jolly 2004). It is made into nine main stages (Figure 4b): **2.1.** Around 16.5 kg of germinated sorghum flour plus 120 l of water are poured into the *elema* inside the beer house. The two ingredients are then mixed with the arm to avoid the forming of lumps. **2.2.** The supernatant, concentrated in the upper two-thirds of the *elema*, is transferred directly into plastic basins (formerly large calabashes), using calabash bowls. The deposit located at the bottom of these large vessels is filtered using a palm-tree leaf basket. The liquid extracted from the spent grains is then collected in plastic basins and mixed with the supernatant previously collected. The spent grains trapped in the filter are kept and dried before being pounded into flour and used in the preparation of daily meals. **2.3.** The mixture of germinated sorghum flour + water that was filtered is then boiled in metal barrels (formerly in *atieda* pots). It is stirred from time to time using a palm-tree branch or a large wooden stick. This phase, carried out in the cooking area, lasts between 8 and 10 h. **2.4.** The mixture of germinated sorghum flour plus water that was filtered and boiled is then cooled in the *elema* inside the beer house. The cooling process lasts 24 h. At this stage, the starch contained in the cereal grains is saccharified. **2.5.** At the same time, 50 kg of ungerminated sorghum grains are pounded using wooden mortars and pestles, and the obtained flour is then sieved. The whole process takes place in the courtyard. **2.6.** The mixture of germinated sorghum flour plus water that was filtered, boiled, and cooled is then filtered using a small basket inside the beer house. The spent grains will be thrown or kept for feeding the animals. **2.7.** 50 kg of ungerminated sorghum flour is mixed with water coming from the rinsing of the filtered spent grains until it becomes a thick paste. This phase takes place in the courtyard. **2.8.** At the same time, the liquid mixture of germinated sorghum flour + water that was filtered, boiled, cooled, and filtered is boiled in the metal barrels (formerly in *atieda)*. After cooking for two hours, the thick mixture of ungerminated sorghum flour + water is added. The two components are strongly stirred together using a palm tree branch or a large wooden stick to avoid the forming of lumps. This phase, carried out in the cooking area, also lasts between 8 and 10 h. At this stage, the wort is ready. **2.9.** The content of the metal barrels is poured into the *elema* inside the beer house and left to cool for 6–7 h.

**Stage 3. The fermentation (1 d)** consists of transforming the fermentable sugars into alcohol using yeast (Jolly 2004). In the Bedik society, the process takes place in three phases (Figure 4c): **3.1.** Around 30 l of the liquid (wort) cooling in the *elema* is transferred to a plastic basin. Two large handfuls of dried germinated sorghum flour are then added, and the mixture is stirred for a long time until it is lukewarm. Once cool, the wort contained in the plastic basin is distributed into the *niene* pots, where active yeasts from previous fermentations have been preserved in the porous inner walls of the ceramic vessels. Fermentation takes place 3–4 h after the wort is transferred to the pots. **3.2.** Between 3–5 l of fermented beer from the *Niene* are then poured into the *elema*. Two large handfuls of dried germinated sorghum flour are added to the contents of these very large vessels. The mixture is stirred until it is homogeneous. Fermentation takes place within 6–7 h. **3.3.** Once fermented, the beer is filtered again before being poured into *niene* or plastic cans (formerly large calabashes with a restricted mouth) and transported for consumption. It is drunk in calabash bowls, or now sometimes in plastic cups.

## Material and Methods

Our research carried out among the Bedik communities followed a well-established method rooted in ethnoarchaeology (David and Carol 2001; Lyons 2017) and drawn from the theoretical frameworks designed by A. Gallay (2011) and Roux and Courty (2016). It fits closely with the comparative technology framework used by O. Gosselain (2017). The primary objective of this approach is to develop reference systems in present contexts driven by archaeological issues. This involves the establishment of systematic connections between material culture and their associated meanings, making the interpretation of analogous archaeological remains easier. Our study uses a direct historical approach (Stahl 2017) insofar as the excavated archaeological site was occupied by the same cultural group as the one studied in the ethnographic surveys.

### Ethnographic Investigations

From this broad theoretical context, our ethnoarchaeological study carried out in 2016 focused on the use of pottery and food recipes of Bedik societies (Mayor and Vieugué in Huysecom et al. 2017). More than 120 ceramic vessels with different functions – including pots used for roasting cereals or tubers, boiling cereals, and sauces, steaming cereals or shea nuts, boiling, cooling, and fermenting beer, storing and transporting water, etc. – were systematically documented. In parallel, another ethnoarchaeological study was conducted, devoted to the vernacular architecture (Pelmoine 2020; Pelmoine and Mayor 2020). Seven Bedik compounds – comprising 55 bedrooms, 10 granaries and 5 beer houses – were precisely recorded. Benefitting from both previous analyses, a comprehensive study targeting the structures and utensils related to beer production in the Bedik country has been undertaken in 2022. 14 beer houses and 157 associated beer pots, as well as several hearths and 6 beer cooking pots, were documented.

The 14 beer houses, selected for study based on voluntary participation of their owner, come from 14 different compounds located in five Bedik villages (Ethwar, Andiel, Iwol, Manda Thiès, and Ninefesha). Eleven of them are from ancestral highland settlements (Ethwar, Andiel and Iwol), while three are established in recent lowland villages (Manda Thiès, Ninefesha). The beer houses from highland *vs* lowland villages could thus be compared to assess their variability in space.

In addition to the precise documentation of the beer houses, several hearths used for beer cooking were informally looked at in three different Bedik villages (Iwol, Andiel, and Ethwar). All of them are from ancestral highland settlements which allows to assess their diversity in this context.

Such a sampling strategy was suitable to provide a first overview of the variability of beer houses and hearths among the current Bedik communities.

#### Architectural Study

The architecture of the beer houses and hearths was the first component investigated insofar as architectural remains are found on most archaeological sites in Africa.

The beer houses were the subject of a detailed study, considering six main criteria that are potentially recognizable in archaeology: (1) The location of the beer house inside the compound, 2) their shape, 3) their size, 4) the building materials and techniques of the walls, 5) the wedging hole of the beer pots, and 6) the presence of an altar. Once these criteria recorded, the total surface area of each beer house was calculated from their diameter. The data collected on the 14 beer houses were finally compared to the ones previously recorded on the 55 bedrooms, 10 granaries, and 5 beer houses (see Pelmoine 2020). Such a comparative approach has allowed us to identify the criteria that might help to distinguish beer houses from other types of buildings such as bedrooms or granaries.

The beer hearths have been more briefly documented as only three criteria were considered: (1) their location, (2) their shape, and (3) their size. Once the observations were made, these hearths were compared to the food cooking areas which were viewed during the documentation of food recipes among the Bedik. We were thus able to establish the criteria that allow us to identify the hearths involved in the beer-making process.

#### Functional Analysis of Pottery

The ceramic vessels related to the beer houses and hearths were the second aspect investigated insofar as potsherds are well-preserved in most archaeological sites in Africa. The 157 pottery vessels coming from the 14 beer houses (except the ritual vessels) and the six found closed to specialized beer hearths were studied from a morphometric standpoint. Five criteria were recorded: (1) rim diameter, (2) maximum diameter, (3) height, (4) capacity calculated from the drawing of the pots, and (5) decoration type. 34 of these ceramic vessels (21%), which are representative of the different types of pots involved in the beer production, were then drawn, and studied using a use-alteration approach. Following previously established methods (Skibo 1992, 2013), use-alteration attributes were characterized macroscopically and classified into three categories: (1) those forming an addition of matter to the surface of the pots (such as soot deposits, charred residues, or lime deposits), (2) those causing a subtraction of matter (like abrasion, chipping, dissolved carbonates, attrition), and (3) those forming a modification of the pottery surfaces (reoxidation, cracks, etc.). The database used for this investigation also considers the location of use-traces along the profile (inner or outer rim/upper body/median body/lower body/base, handles, and decorative elements) as well as their morphology (punctual, covering, linear, ring-shaped). Once recorded, the typometry and use-traces of the 157 beer pots were finally compared to the ones from previously published studies (Arthur 2002, 2003; Mayor and Vieugué in Huysecom et al. 2017). Such a comparative approach has allowed us to determine the criteria that are diagnostic of beer pots.

### Archaeological Excavations

In addition to the ethnographic study, we carried out archaeological surveys in the region to identify an abandoned village suitable for excavations. We looked for a site accessible, and located in a flat area where erosion was not too intense and archaeological layers and remains were potentially well preserved. We decided to excavate a recently abandoned ruined Bedik village called Eguong, after obtaining the agreement of Jean-Baptiste Syaro Keita of Iwol, and the descendants of the former inhabitants in Andiel and Mangama. According to local memory, the village of Eguong was established close to the village of Iwol (≃ 1 km), well before the war with Alfa Yaya at the end of the nineteenth century. It was abandoned progressively around the Second World War. Only one compound inhabited by a family remained occupied until 2009, and the patriarch (patronym Sadiaxu) was a major religious chief (oral communication J.B. Keita). The 2022 archaeological investigation focused on this compound. The ruined buildings and hearths were still visible and functionally identified by J. Keita before any excavation took place. According to him, the compound would have been composed of six bedrooms, two granaries, one beer house, a courtyard, and exterior domestic hearths (for cooking inside the compound and for beer production at the outskirts), as well as disposal areas and a collective place of palaver at the periphery. Two bedrooms, one granary, and the beer house were excavated, while test pits were carried out in the center of the courtyard, two outdoor hearths (including the hearth for cooking beer), the place of palaver, and two disposal areas. A total of 49 m² were excavated. In most cases, the archaeological layer was thinner than 30 cm.

#### Architectural Study

At Eguong, the different buildings and cooking areas investigated were excavated stratigraphically using the opposite square method. A 21 m² area was implemented to encompass most of the beer house and its immediate surroundings. However, the indoor and outdoor altars were not uncovered, in compliance with the wishes of the inhabitants of Iwol. A 1-by-1 m test pit was also implemented at the center of the beer hearth to evaluate the state of preservation of this installation. To allow comparisons, the architecture of the different buildings and cooking areas excavated at Eguong was documented using the same approach as the one used in ethnography.

#### Functional Analysis of Pottery

2051 sherds were uncovered during the excavation of the Sadiaxu family’s compound, but only 18 ceramic vessels could be reconstructed. 116 sherds were uncovered inside the beer house, contributing to 10 of the reconstructed vessels, while no sherds were found in association with the beer hearth. The archaeological pottery was studied similarly to the ethnographic one, using morphometrical and use-alteration criteria, although the method was adapted to the fragmentation of the archaeological assemblage by extrapolating from the sherds the rim diameter, maximum diameter, and height.

## Results

### Location and Architecture of Structures Linked to Beer Production

#### Ethnographic Data

##### - Beer Houses

**Figure 5.**
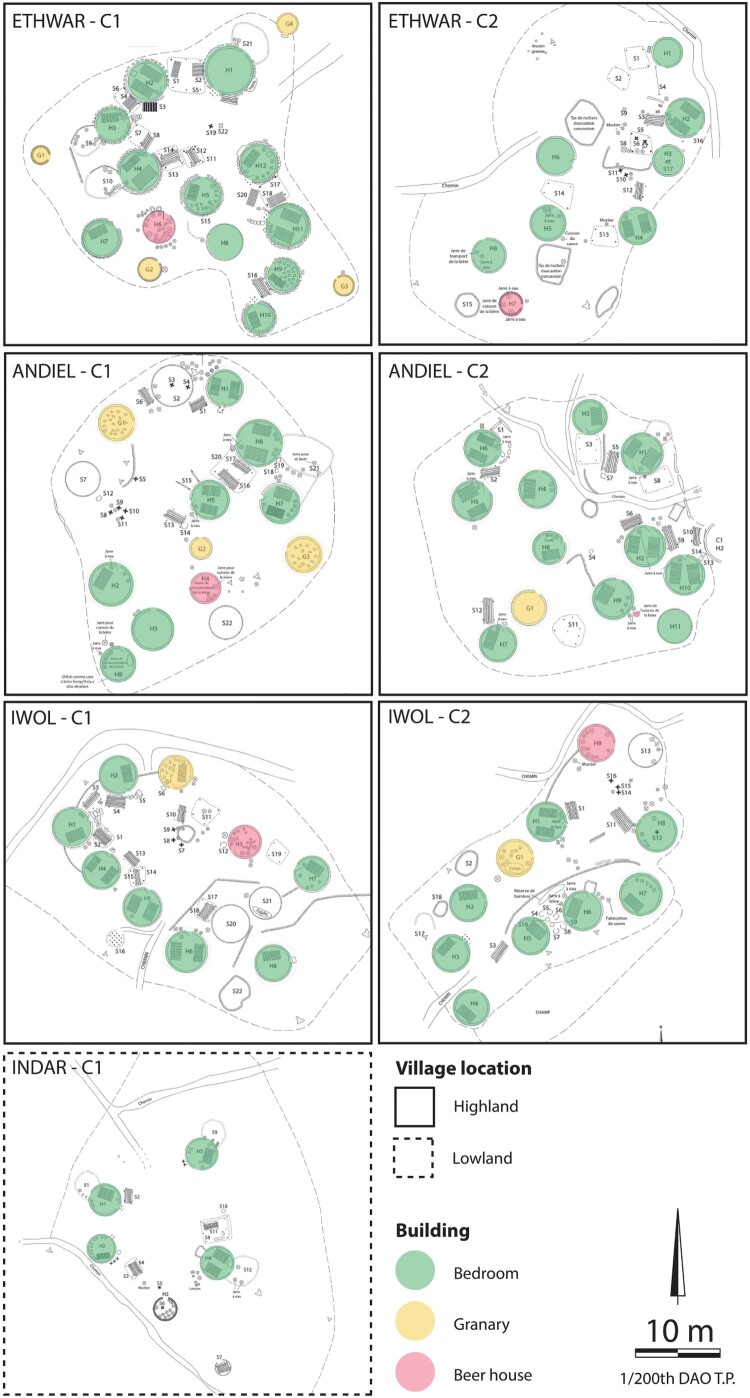
Location of beer houses inside several Bedik compounds and shape of the buildings (after Pelmoine 2020, modified).

In the Bedik country, the beer houses are loosely positioned around the central courtyard of the compound, in the same way as the bedrooms and the granaries (Figure 5). Thus, the location of the buildings is not a relevant criterion to identify their function.

All the beer houses documented are round (14 of 14), like bedrooms (55 of 55) and granaries (10 of 10) (Figure 5). The shape cannot be used to distinguish the beer houses from other types of buildings.

**Figure 6.**
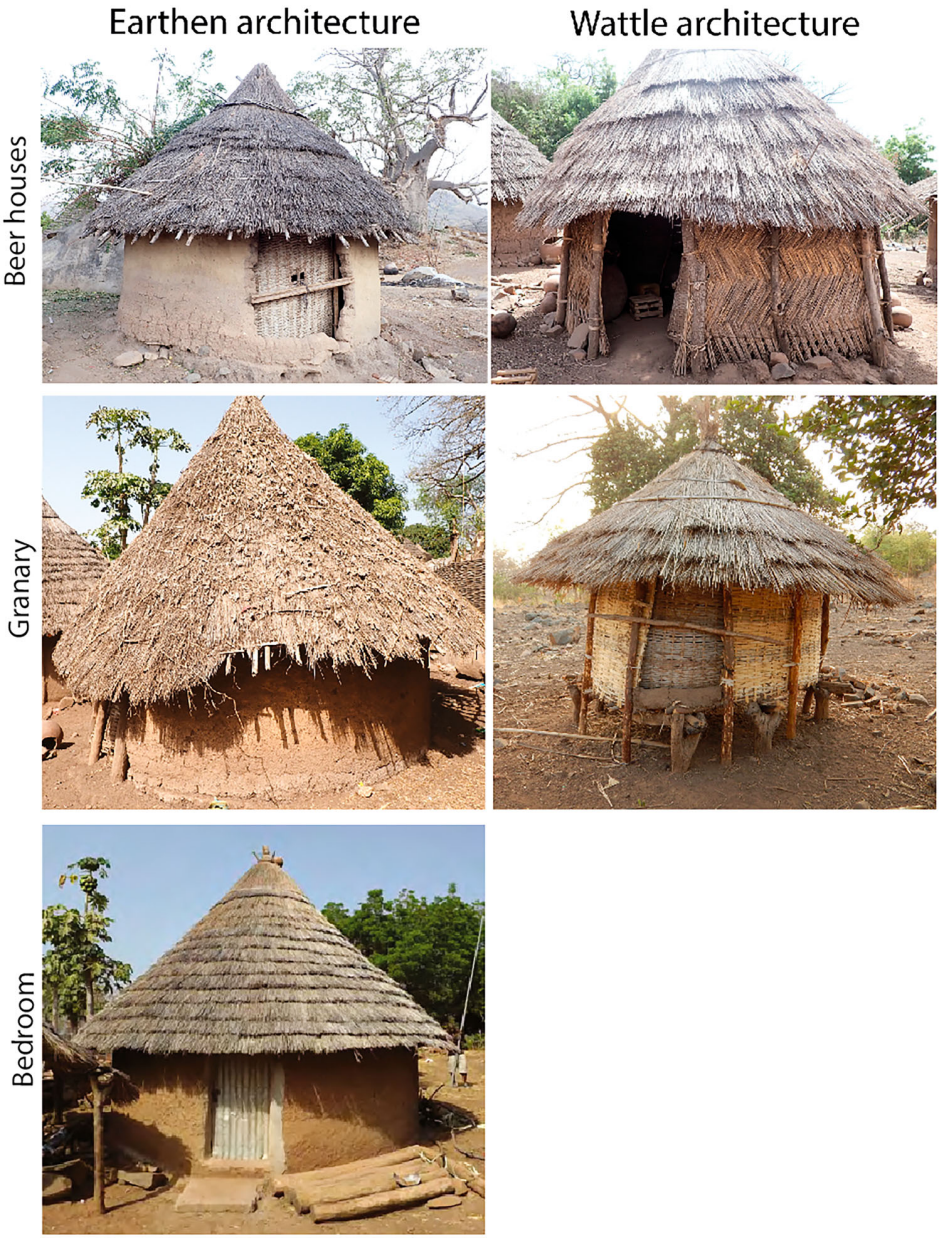
Building materials and techniques of beer houses (top), granaries (middle) and bedroom (bottom).

Most of the beer houses are made of earth walls, built using the cob technique (11 of 14), but some of them are also made of perishable plant material walls (either bamboo or grass), built using the wattle technique (3 of 14). Like beer houses, granaries are constructed using an earthen wall, and are rarely made with vegetal materials and the wattle technique (3 of 10). Only bedrooms are systematically made of earthen walls, built using the cob technique (Figure 6) (Pelmoine and Mayor 2020). The building materials and techniques are not too informative about the function of the buildings.

The recorded beer houses vary in size, ranging from 3,10 m to 4,84 m in diameter (7.55–18.40 m^2^) with a median at 3.54 m (9.85 m^2^). They tend to be interspersed between the small granaries measuring from 2.10–2.61 m (3.45-5.35 m^2^) and the large ones from 3.80–4.05 m (11.35-12.85 m^2^). The beer houses appear furthermore to be slightly smaller than the bedrooms, the diameter of which ranges from 3.33 m to 5.20 m (8.55–21.22 m^2^) with a median at 4.20 m (13.85 m^2^) (Figure 5; Supplement 2). The extraordinary size of one beer house (Andiel – Syaro Keita; 4.84 m diameter) can be explained as it is a reassigned building that was once a bedroom. To some extent, the dimensions of the buildings seem to be a diagnostic criterion to differentiate the beer houses from the other types of buildings.

All the beer houses (14 of 14) have shallow pits lined with stones supporting the pointy base of large vessels used to germinate grains, cool and store malt, and ferment beer (14 of 14) (Supplement 3). This differs from bedrooms and granaries where pots used to store foodstuff or water are either put directly on the ground or maintained using wooden posts. The presence of wedging holes with stones can be another diagnostic criterion for identifying beer houses.

Some beer houses (6 of 14) also include an altar in the form of grouping of stones, sometimes accompanied by specific ritual pots. Such a particular feature, which is most often located inside the building, is totally absent in the bedrooms and the granaries of the Bedik villages. Its presence inside a building may therefore indicate a beer house.

##### - Beer Cooking Areas

The hearths related to beer production are always located on the outskirts of the compounds, while the ones used for cooking food are consistently located inside the courtyard. The location of the fireplaces can be considered as a criterion to distinguish the former from the latter. However, caution must be taken because the hearths used for beer production can sometimes also be used for soap making, which requires the same use of large vessels remaining on the fire for several hours or days.

The hearths related to beer production are composed of three large stones used to stabilize the large vessels, sometimes fitted with sherds. The stone structure is always associated with an ashy patch. The hearths used for food cooking are very similar (Supplement 4) even if they tend to be a bit smaller insofar as smaller pots are placed there. No obvious criteria allow us to distinguish them.

In short, the ethnographic study shows that only three criteria can help differentiate beer houses from other buildings: their size, smaller than bedrooms and large granaries and bigger than small granaries, the presence of wedging holes, and the presence in some of them of an altar. Only one criterion clearly permits to distinguish hearths related to beer production from the ones related to food cooking: their location outside the compound.

#### Archeological Data

##### - Beer House

**Figure 7.**
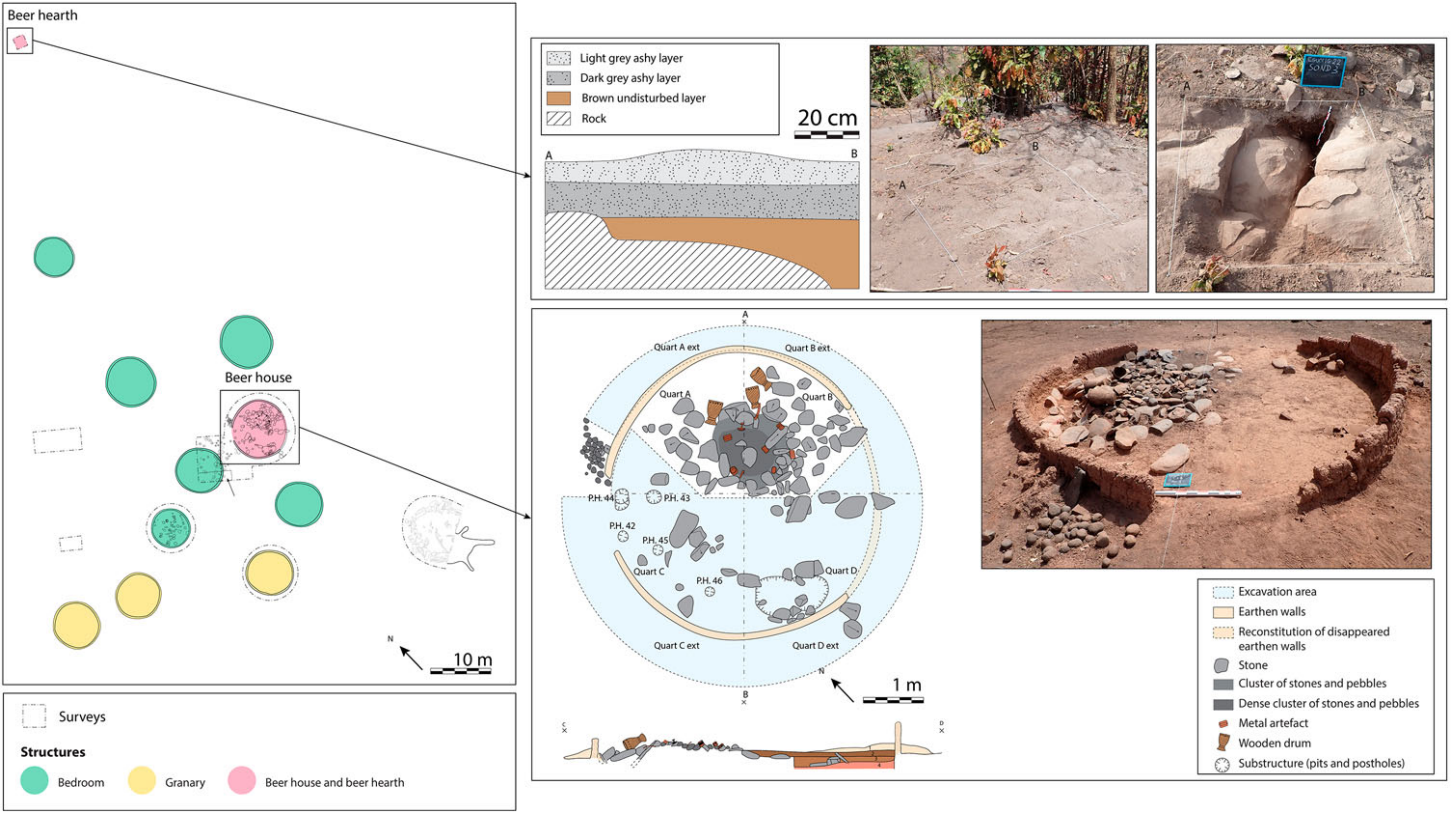
Plan and section of the beer house and cooking area excavated at the abandoned village of Eguong.

The beer house uncovered at Eguong is loosely located around the central courtyard of the compound, alongside bedrooms and granaries. It is round-shaped, as are the other buildings still visible at the surface of the site (Figure 7). Its diameter, which is about 4,70 m (17.35 m^2^), is surprisingly large compared to the bedrooms which range from 3.56–4.78 m (9.95-17.95 m^2^) and that of the granaries which measure between 4.06 and 4.20 m (12.95-13.85 m^2^) (Figure 7; Supplement 5). The beer house excavated at Eguong is made of earth walls, most probably built using the cob technique, such as the three other buildings investigated. Two shallow pits of 40 and 30 cm in diameter, fitted with stones, were recognized along the wall in the southern quarter of the beer house (Figure 7). Such structures, which likely correspond to the wedging holes for the large beer pots (*elema*), were not identified in the two bedrooms or the investigated granary. Several post-holes dug in the substratum, were surprisingly identified at the entrance of the beer house, although it was not possible to determine whether they were part of a layout inside the beer house or linked to earlier occupations. A much larger number of post holes, between 4 and 20 cm deep, were recognized in the two bedrooms excavated but none was identified in the granary. However, it should be noted that the substratum was brittle in this area, making the identification of post holes difficult. The most prominent feature of the beer house is the altar structure, taking roughly 40 percent of floor space and composed of large slabs on the outside layers and smaller module stones toward the center, as well as metallic elements, and two wooden drums. Such a particular structure was totally absent in the two bedrooms and the granary.

The presence of two shallow pits and one altar inside the excavated building are compatible with oral tradition according to which it would have been used as a beer house. On the other hand, the size of the structure does not match with it, being larger than expected for such a building.

##### - Hearth Used to Cook Beer

The hearth used for making beer is located outside the compound investigated at Eguong, quite far from the buildings (about 30 m to the north), while the one used for cooking food is in the courtyard, close to the granaries (about 10 m to the north) (Figure 7). The former consists of a thick layer (about 20 cm) of gray ashy sediment that was visible on the surface whereas the latter displays a thinner deposit of ash. The three stones typically used to stabilize the large cooking vessels (*atieda*) were not uncovered for the hearth related to beer production unlike the one used for food cooking (Figure 7). They may have been removed for reuse in the neighboring fields still cultivated by the inhabitants of Iwol.

### Pottery Associated with Beer Production

#### Ethnographic Data

##### - Pottery Found Inside the Beer Houses

Three main morpho-functional groups of pottery are present inside the beer houses of the different Bedik villages (Andiel, Ethwar, Iwol, Manda Thiès et Ninefesha). However, their frequencies vary greatly (Supplement 6).

**Figure 8.**
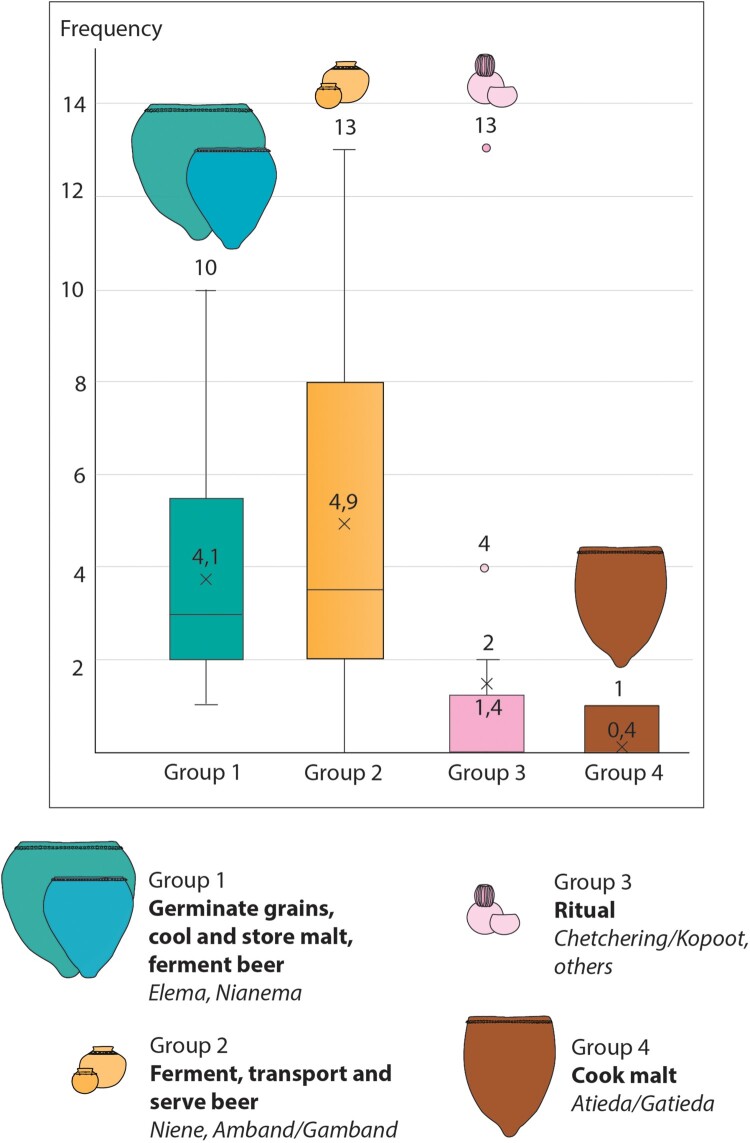
Frequency of the four functional groups of pots found inside beer houses.

(1) The large vessels used to germinate grains, cool and store malt, and ferment beer (*elema, nianema*) are the only functional type that is systematically found inside the beer houses (52 vessels in total), although they can vary from one to 10 individuals (median at four) (Figure 8). They are placed inside, along the walls of the beer house.

**Figure 9.**
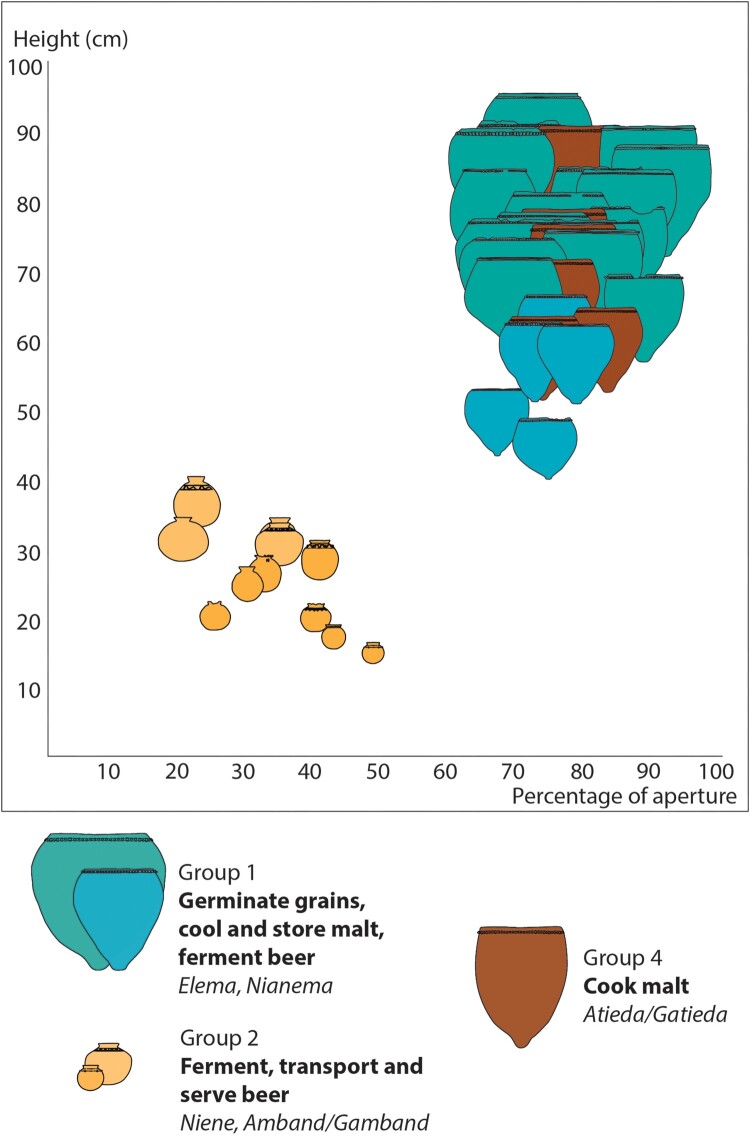
Morphometry of the three main functional groups of pottery related to beer-making.

They are conical shaped, with a thick pointy base, suitable for sinking into the ground and supporting large volume of liquid. Their mouth width varies from 38.4–78.4 cm (median at 54 cm), their maximum diameter from 51.6–99.1 (median 71 cm). Their volume ranges approximately from 65 to 360 l (median 174.9 l). The rim is often decorated using a coil with finger impressions (51 of 52 pots). Subgroups of this pottery type (*nianema)* have been occasionally observed (6 vessels in total) and were only found in three beer houses, one of them having yielded four vessels. They are similar to the large vessels (*elema*) from a morphological point of view, although they are smaller, between 30 and 90 l (median 64.5 l) (Figure 9).

**Figure 10.**
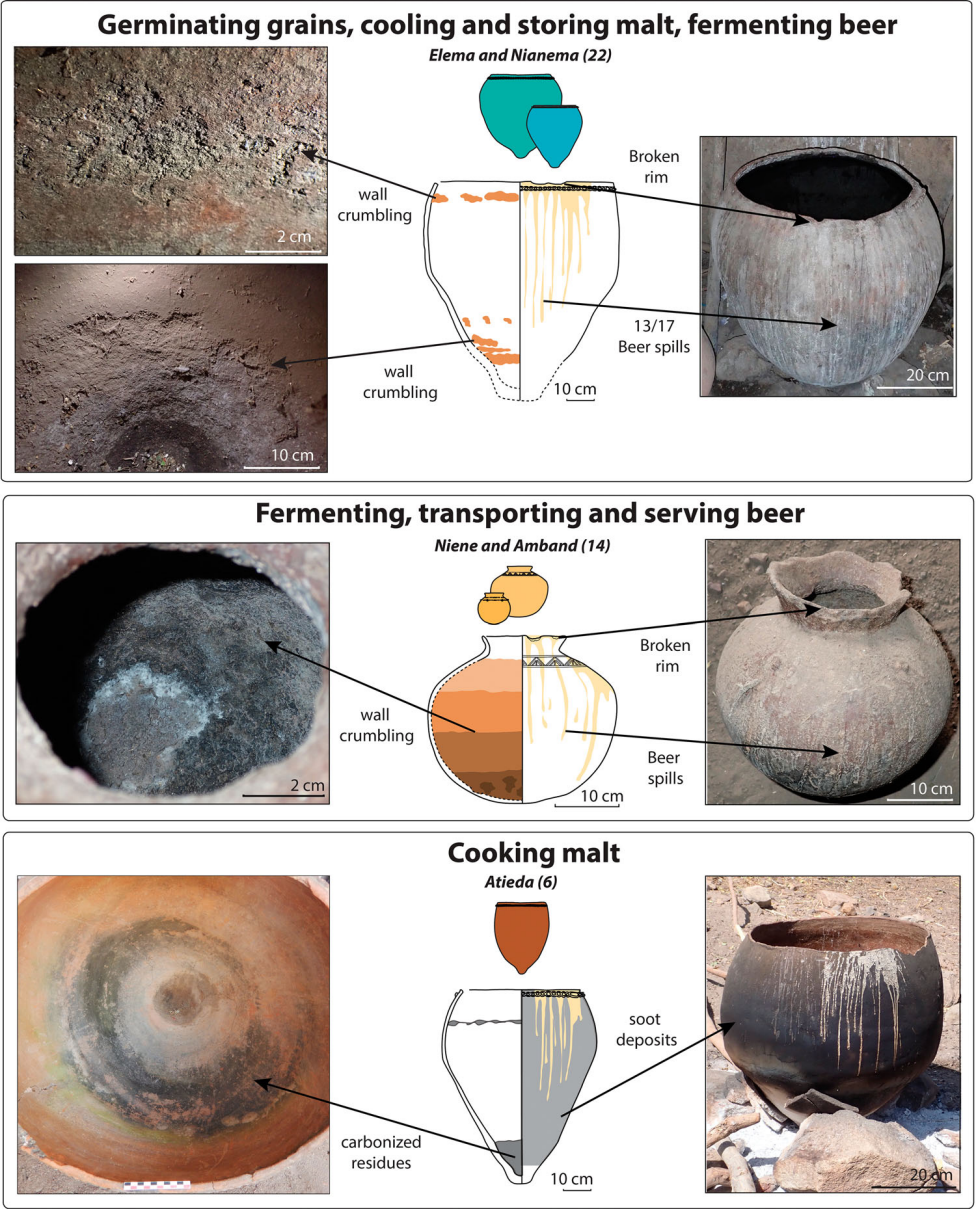
Typical use-alteration of the beer pots.

Large vessels used to germinate grains, cool and store malt, and ferment beer (*elema*) display an over-representation of beer spills and inner attrition in a horizontal fashion under the rim (10 of 17 pots) and/or in the lower body (8 of 17 pots). Based on their position, the attrition could be induced by the physico-chemical reactions with the corrosive foam and the fermentation. They can be accentuated and/or accelerated by the stirring motions with wooden sticks. They also display a high proportion of broken rims (10 of 17 pots), which the owners claim happens when a container full of liquid is rested on its rim during the different transfer operations. The smaller versions of this functional type (*nianema*) display similar beer spills (2 of 5), broken rims (1 of 5) and inner attrition (2 of 5, located on the upper and mid body) (Figure 10).

The pots used to germinate grains, cool and store malt, and ferment beer (whether they are *elema or nianema*) clearly differs from other functional classes of ceramic vessels, both by their shapes (conical profile, slightly closed opening, pointy base, capacity most often over 65 l) and their use-traces (numerous outer beer spills, inner attrition) (Mayor and Vieugué in Huysecom et al. 2017). They can therefore be easily recognizable within the pottery assemblages.

(2) The vessels used to ferment, transport, and serve beer (*niene, amband*) are the most common types (69 documented in total). Up to 13 individuals were observed in a single beer house. However, this type of beer pot can sometimes be completely absent inside a beer house which can be explained as the pots are mobile and often lent to other family members or acquaintances. They are usually found near the *elema* and are often grouped (Figure 8).

They are globular shaped with a very narrow opening and sometimes a bottle neck. Mouth width ranges from 5.4–11.8 cm (median 7.5 cm) while the maximum diameter is between 15.6 and 36 cm (median 24 cm), so the rim diameter represents between 20 and 48 percent of maximum diameter (median at 33.8%). The volume of the documented ones is between 2.1 and 21.5 l (median 9.9 l). They can be plain (38 occurrences out of 58 *niene*) or display decorations such as geometric incisions, grooves, impressions, and added clay lumps (20 of 58) (Figure 9).

The pots used to ferment, transport, and serve beer (*niene*, *amband*) display beer spills (13 of 14), broken rims (9 of 14), and severe attrition, either in the inner upper body (4 of 14 pots) or incrementally on the entire inner wall except the rim part (7 of 14 pots). As for elema, the fermentation process, as well as the friction of the small stirring wooden sticks on the pottery walls, may be the combined cause of the use-alteration (Figure 10).

These pots are different in terms of shapes (globular profile, very closed mouth, round base, small capacity most often lower than 20 l) and use-traces (numerous outer organic spills, inner attrition), compared with other functional classes of ceramic vessels (Mayor and Vieugué in Huysecom et al. 2017). They can, therefore, be identified.

(3) The pots used during rites are present (from one to 13 in number) as soon as there is an altar inside the beer house. They are stored over the stone altar. The beer house that delivered the most numerous ritual pots belongs to a Kamara family who is responsible for ceremonies (Figure 8).

Neither morphometric nor use-alteration analyses of these specific pots were carried out according to the wish of the owners. However, we were able to observe that they appear in the form of various morphotypes, including bi-lobed pots with a very restricted opening and highly decorated body with added clay lumps, called *chetchering* in Biwol and *kopoot* in Banapas *(*Supplement 3). Each altar and spirit need specific forms and decors, hence their diversity. Such ritual pots clearly stand out from the other morpho-functional classes of ceramic vessels in the Bedik country. They can therefore be easily recognizable within the pottery assemblages.

##### - Pottery Associated with the Hearths

Only one morpho-functional type of pottery is found in association with the hearths used for beer making. These are pots used to cook the malt (*atieda*) which exist today only in a quite limited number. Often found in one copy per fireplace, they are either placed upright on the three stones of the hearths or placed upside down next to it.

Their shape, size, and volume are quite like the large ceramic vessels used to germinate grains, cool and store malt, and ferment beer (*elema*). *Atieda* are conical shaped with a thick pointy base. The rim diameter greatly varies between 38.4 and 78.4 cm (with a median at 48.3 cm) while their maximum diameter ranges from 51.6–99.2 cm (with a median at 62.7 cm). Their capacity is between 66 and 332 l (with a median at 114 l) (Figure 9).

The vessels used to cook the malt (*atieda*) show different use-traces compared to that of the large pots used to germinate grains, cool and store malt, ferment beer (*elema*). They always display outer soot deposits and inner charred residues (6 of 6), as well as beer spills (6 of 6). Only one pot shows some inner clay wall attrition. The scarcity of such use-alteration may be explained by the fact that the fermentation does not take place inside the cooking pots (Figure 10).

Here again, the large pots used to cook malt (*atieda*) clearly differ from other functional classes of ceramic vessels, both by their shapes (conical profile, slightly closed opening, pointy base, large capacity over 60 l) and use-traces (numerous organic spills, outer soot deposits, inner charred residues) (Mayor and Vieugué in Huysecom et al. 2017). They can, therefore, be distinguished.

According to our study, three main criteria can be used to differentiate beer pots from other functional classes of ceramic vessels: (1) The presence of a thick pointy base; (2) A large capacity (3) The presence of attrition on the inner surface of ceramic vessels.

#### Archaeological Data

##### - Pottery Found Inside the Beer House

**Figure 11.**
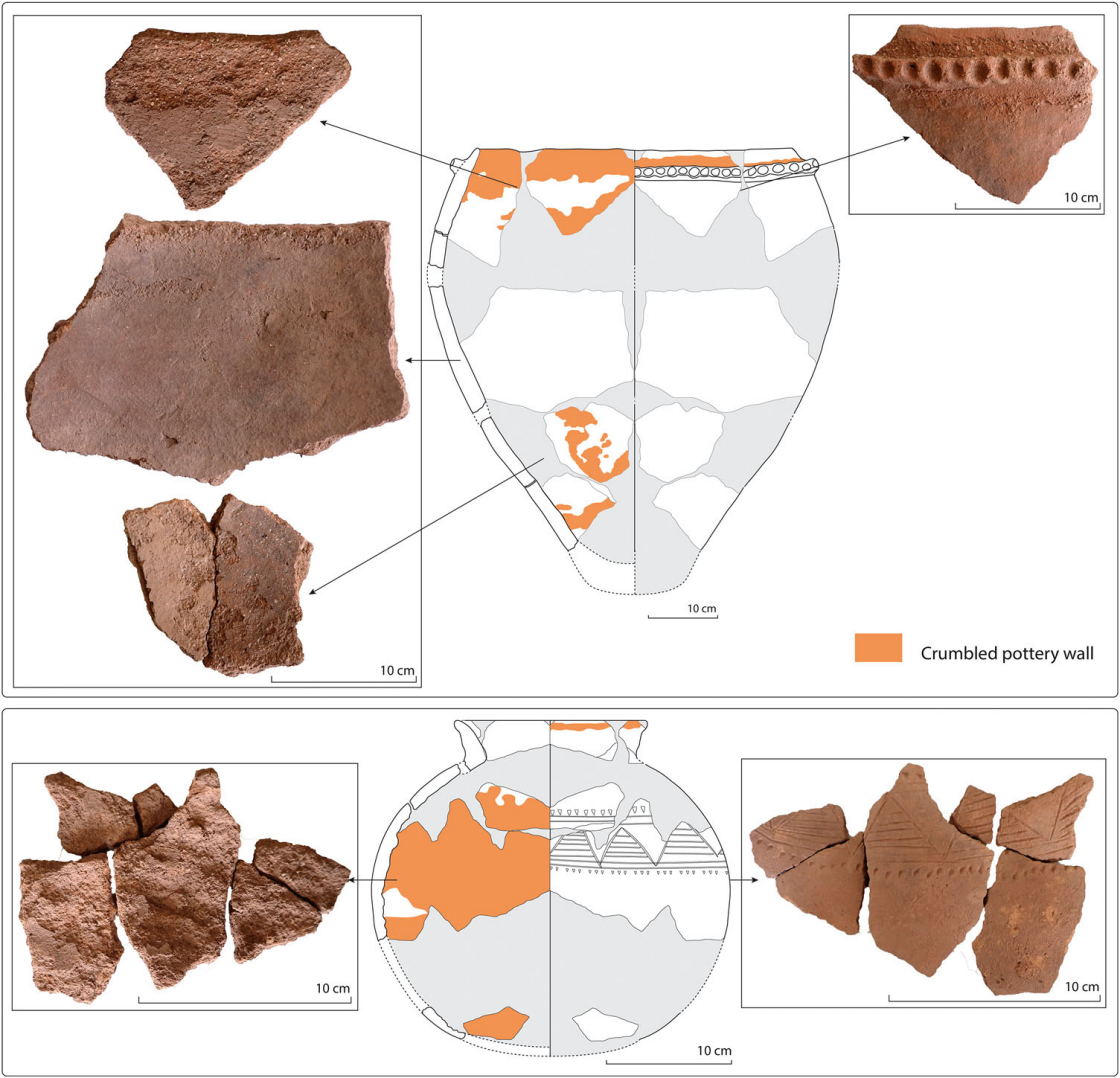
Large and small vessels from the abandoned village of Eguong displaying inner wall attrition.

At Eguong, several large fragments of ceramic vessels were uncovered inside and outside the beer house, at the surface, and a few centimeters below. The precise number of such large vessels could not be established but may be between three and six individuals. One individual was found complete but broken into large sherds found inside and in the immediate surroundings of the beer house at the surface. It is interpreted that the pot was broken during its removal from the beer house, probably with the purpose of being reused in another house. No sherds from this type of pottery were found in the refuse areas. Some sherds display attrition on their inner surface (Figure 11). On the most complete pot, they are localized in a horizontal fashion below the interior rim and in spots in the lower inner body. Based on the large size of the pots and the presence of interior attrition, we can assume that these large vessels were *elema* used to germinate grains, cool and store malt, and ferment beer.

Only one small globular vessel with a restricted opening was discovered inside the beer house, right under the surface. The pot is decorated with a set of incised triangles and a horizontal line of punches. Almost all its sherds display a severe interior attrition whereas they show no use-alteration on their outer surface (Figure 11). The rim also shows traces of chipping. From a morphological and use-alteration point of view, this pot is analogous to *niene* used to ferment, transport, and serve beer.

##### - Pottery Associated with the Beer Cooking Area

At Eguong, no pottery sherds were found associated with the beer hearth.

### Synthesis

By comparing the ethnographic and archaeological data collected on the buildings and cooking areas of several Bedik villages, we were able to establish the relevant criteria for identifying the structures related to beer production within ancient settlements.

#### Identifying the Beer Houses

Our study reveals the difficulty in identifying beer houses solely based on architectural remains, even though the Bedik country features exceptional specialized structures. The location and the shape of the buildings, as well as their wall-building materials and techniques, do not allow us to differentiate them from other structures. In both the current village of Iwol and the abandoned site of Eguong, the beer houses are located among other buildings. All of them are round-shaped, like bedrooms and granaries. Most are made up of earthen walls using cob techniques, similar to other buildings. The size of the buildings is not a relevant criterion either, as the archaeological beer house uncovered at Eguong is significantly larger than all the ethnographic beer houses recorded at Iwol. It is also bigger than the bedrooms and large granaries of the compound excavated. This can be explained as the owner of the Sadiaxu family’s beer house at Eguong was the most important religious chief of the region and likely chose to build a large beer house to accommodate a large altar inside it. Apart from this altar, the presence of shallow pits surrounded by stones alongside the walls of the buildings is the only diagnostic criteria to differentiate the beer houses from other structures. Two such pits were recognized during the excavation of the Sadiaxu family’s beer house at Eguong, suggesting the presence of at least two large vessels used to germinate grains, cool and store malt, and ferment beer (*elema)* inside it. Other pits might not have been identified during the excavation, due to their shallow depth. Nevertheless, the available space suggests that the beer house at Eguong could have fitted 6 large vessels at the most.

The ethnoarchaeological investigation underscores the high significance of pottery remains within the buildings as a clue to identify beer houses in archaeological contexts, although their potential obviously depends on their state of preservation. A limited number of pots was uncovered inside the Sadiaxu family’s beer house at Eguong (4 pots) compared to that of the Keita’s and Kamara’s family beer houses at Iwol (10–21 pots). Such a difference can be explained as the complete vessels were likely taken away at the time of the abandonment of the village. Nevertheless, some fragmentary sherds were left inside (116 sherds). At least three large, thick pots with a finger impressed coil near the rim and a pointy base, as well as one small and thin vessel with a very restricted opening decorated with incisions and impressions on the upper body were thus recognized. Most of them show severe attrition on their inner surface. The typometry and use-alteration of the ceramic vessels found inside the Sadiaxu family’s beer house at Eguong are therefore compatible with *elema* and *niene*-type pottery.

#### Identifying the Beer Cooking Areas

Our case study shows that the identification of the beer cooking areas based only on the architectural remains is also illusive. The shape and the size of the hearths is not a strong diagnostic criterion to distinguish the ones used to make beer from the ones used to prepare food. Both in Iwol and Eguong, they are materialized by a thick layer of ash sediment, sometimes associated with three large stones. Only the location of the hearths seems to be relevant to differentiate the ones related to beer production and the ones linked to food preparation. Whether in Iwol or Eguong, the former are in the outskirts of the compounds while the latter are in the courtyard.

The ethnoarchaeological observations carried out on the cooking areas of several Bedik villages show that pottery remains are not very informative for identifying the hearths used to boil malt. At Eguong, no sherds were identified in association with the excavated hearth linked to beer production. This is not very surprising given the limited number of *atieda*-type pots found close to the hearths in Iwol, often re-used as chicken coops. Such a complete absence in Eguong can be explained as the complete beer cooking vessels were likely taken away, and the sherds of the broken ones disposed of in dumping zones far from the compound.

## Discussion: Exploring Cross-Cultural Issues

To find out if the criteria for identifying beer production among the Bedik were also relevant in other contexts, we carried out a preliminary cross-cultural analysis. This approach explores the potential applications of our results to other African societies, by highlighting common patterns and mechanisms across diverse chronological and spatial contexts. To do so, we have compared our data with those coming from various cultural groups in Africa. Unfortunately, the available data published so far on the structures and vessels linked to the manufacture of this fermented beverage in Africa have proven to be extremely fragmentary. Only a few studies, including those carried out among the Dogon in Mali (Bedaux 1986; Gallay, Huysecom, and Mayor 1998, 2012; Jolly 1995, 2004), the Hidé (Cameroon) (Eguchi 1975), the Gamo (Ethiopia) (Arthur 2002, 2003, 2021) or the Zulu (South Africa) (Fowler 2006), provide useful information on the topic. The structures associated with beer-making are often described only briefly, whether in terms of location, shapes, and sizes, or building materials and techniques (see Dueppen and Gallagher 2021; Eguchi 1975; Jolly 1995, 2004; Müller-Kosack 2003; Seignobos 1982; Van Beek 1978). Also, the pottery containers involved in the production of beer are only briefly presented using simplified generic types, without detailed morpho-dimensional analysis (except Fowler 2006) or use-alteration studies (except Arthur 2002, 2003). Consequently, we gathered extremely partial and heterogeneous comparative data. Despite these limits, this approach has allowed us to identify a first set of diagnostic criteria for identifying this fermented beverage across cultural borders.

### In West Africa

#### The Dogon Country (Central Mali)

Beer production in the Dogon Country has been extensively investigated by the anthropologist E. Jolly (1995, 2004), who analyzed the social significance of this fermented beverage in this society. To a lesser extent, it has also been documented by the ethnoarchaeologists R. Bedaux (1986), and A. Gallay, Huysecom, and Mayor (1998, 2012), who studied pottery traditions in the region.

In the Dogon country, beer-making takes place in several locations. Germinated cereals are ground either in the public squares of the villages or in the courtyards of the compounds; malt is cooked either in shelters in front of the quadrangular houses or in kitchens (especially for the preparation of ritual beer) (Jolly 2004, 274). Not all compounds feature a place to brew beer (Jolly 1995, 182).

The hearths used to cook malt are built at the foot of house walls, designed to refract heat (Jolly 2004, 59). They are made of large stones or mud feet, planned to set three or four large cooking vessels depending on the region (Jolly 1995, 181). They are like the ones used to cook food but have larger feet or stones. Fermentation jars are often wedged in the ground.

Jolly (1995, 187) also specifies the different types of pottery used to make beer, however without considering the different traditions (see Gallay, Huysecom, and Mayor 1998): (1) hemispherical fermentation jars, large with very wide openings (volume 100–200 l). According to A. Gallay (Gallay et al. 2012, 188), these are the largest pottery, both in height (43-48 cm) and maximum diameter (58-62 cm); the more variable opening diameter (32-62 cm) overlaps pots used for water storage. (2) cooking jars, spherical, slightly ovoid, with a slightly closed opening (25–35 l); (3) pottery for transporting or serving beer, of medium size and with a slightly closed opening, into which beer is drawn with a calabash. There are also narrow-necked pots used to pour beer directly into drinking calabashes. In the village of Diallassagou (Tradition Dogon C), these pots (between 33–66 cl) are used to measure beer for sale; (4) Ritual pots containing a man’s share of the beer, 3–4 l, or an ancestor’s, smaller and more open. They are placed in a small room in the house and filled with beer every year; (5) Libation dishes (4-5 cm in diameter), embedded in a wall or in the clay of an altar, in which offerings of food and unfermented beer are deposited. Archaeological excavations in the rock shelter of Dangandouloun have evidenced that similar vessels date back to the seventh century AD (Mayor 2011, figures 100, 111, 113, 126). In Tireli (tradition Dogon A), R. Bedaux (1986) describes two groups of beer-making pottery. The first group comprises spherical pottery of varying sizes, with a wide opening. Three vernacular terms are used to distinguish them: first, those for “cooking” beer; second, those for “storing” beer; and third, those for “storing or fermenting” beer. The second group comprises narrow-mouthed spherical pottery of varying sizes, with three vernacular terms distinguishing the types, all related to the “storage” of beer.

#### The Mouhoun Bend (Mouhoun Province, Burkina Faso)

Dueppen and Gallagher (2021) briefly documented the production of beer in Tora, in the Mouhoun Bend.

The authors do not mention the presence of specialized buildings linked to beer-making. Malt seems to be cooked and brewed in the open air. They only mention the use of permanent installations to cook malt, which consists of earthen brick structures to elevate the pottery vessels above the fire. The fermentation is processed in heavy vessels that are embedded in the ground in a permanent setting.

According to this study, three types of pottery are involved in the production of beer: (1) Cooking vessels with an open mouth and a slightly restricted neck, and a flared rim. They tend to be larger than cooking pots used for food, except those needed to prepare large batches of millet porridge. (2) Large brewing vessels that are open and unrestricted, with thick walls and coarse fabric. (3) Small open bowls used to cultivate the yeast.

### Beyond West Africa

#### The Mandara Mountains and the Highlands (Northern Cameroon)

Several ethnographic studies documenting the production of beer in the Mandara mountains have been carried out by P.K. Eguchi (1975), W.E. van Beek (1978, 2002), C. Seignobos (1982), and G. Müller-Kosack (2003).

In this region, the spatial distribution of beer production stages varies among cultural groups (Seignobos 1982). Hut-breweries and kitchen-breweries are commonly mentioned, even if it remains often unclear whether wort boiling occurs inside these structures or in the adjacent courtyards. Scarce information exists on hearth morphology (Seignobos 1982). Moreover, among the Kapsiki, beer production by men for ceremonies is distinct from brewing by women for domestic and market-oriented purposes, resulting in complex multiple brewing spaces within the compound (Seignobos 1982, 105; Van Beek 1978).

In most cultural groups, beer production occurs in specific buildings, either completely dedicated to this activity (hut-brewery) or only partially (kitchen-brewery), but also in adjoining courtyards. More precisely, in Mofu kitchens, a hearth larger than the ones for food preparation is used for brewing beer inside the buildings (Seignobos 1982, 42), while the Mafa may set up a courtyard hearth near the brewery-kitchens during the dry season (Seignobos 1982, 78). In most of the cultural groups, brewing spaces are typically located inside the compound, though exceptions exist, such as the Koma who install a three-jars wort-cooking apparatus outside the village, near a stream where the millet is immersed in closed baskets before being put to germinate (Seignobos 1982, 162), or such as the Hide who brew beer in their terraced fields (Eguchi 1975).

Compounds also dedicate specific areas to other stages of beer production, such as the Mafa drying sprouted sorghum near the goat shed (Müller-Kosack 2003) or the Hide storing beer for rituals in the ancestors’ hut and serving it in the courtyard in front of it using calabashes (Eguchi 1975). In terms of size and building materials, brewery huts are not consistently distinguished from other buildings. The internal layout depends on the building’s nature, with kitchens characterized by grinding tables, hearths, and various ceramics including beer jars, while brewery huts feature large brewing jars, often buried in the ground (Kapsiki) and smaller ones used for transport (Seignobos 1982, 106).

In the Hidé society, Eguchi (1975) notes three distinct types of pottery related to beer-making: (1) Large jars, measuring 70 cm in height and 60 cm in diameter, are used for brewing malt and fermenting beer. (2) Medium-sized jars with round bases and narrowed necks, measuring 15–20 l in capacity, are used for storing beer near the altar that is in the ancestor’s hut. (3) Small, tightly sealed 3-to-4-l jars are used for serving beer. Among the Kapsiki (Van, Beek 2002), large jars for brewing beer and small narrow-necked jars for cooling and fermenting the beverage are mentioned. In a Mafa community, Müller-Kosack (2003) shows a photograph of pottery with a round base and a very narrow opening, designed for fermenting and serving beer. Among the Mofu and Giziga, jars for “preparing” beer are indistinguishable from those used for storing water. Both display variable shapes and sizes, some of them having large dimensions (approximately 90 cm in height, 60 cm in diameter and 50 cm in internal opening diameter). Jars used for transporting beer are described as being like the ones used for transporting water. They usually have very closed shapes (e.g. 47 cm high, 42 cm diameter, and 8 cm in internal opening diameter) (Barreteau and Delneuf 1990).

#### The Gamo (Ethiopia)

J. Arthur documented the beer-making among the Gamo (Arthur 2002, 2003, 2021, 2022).

Limited information has been published regarding the structures dedicated to this activity. It is nevertheless mentioned that beer processing occurs within specialized kitchens and storage buildings (Arthur 2003, 523), which can only be found in the wealthiest households.

More detailed data are available concerning the pottery assemblages. According to Arthur’s study, two main types of pottery are used to produce and drink beer: (1) Large jars (N = 52 jars; volume = 33.6 l on average, 2.8 l minimum, 124.7 l maximum) that are globular shaped, have a restricted opening and a neck (Arthur 2003, 522). They seem to be used both for cooking the malt and fermenting. The author specifies that jars are made into five different sizes of which the largest is used for beer; (2) A special size jar, smaller than the previous one, is used specifically to transport beer into the field. Based on the use-alteration study of these vessels, J. Arthur has highlighted the regular presence of attrition on their inner surface. He was thus able to demonstrate the link between this specific type of use-alteration attribute and fermented beer (Arthur 2002, 2003), whatever the type of clay exploited for the manufacture of pottery (Arthur 2003, 524).

#### The Zulu (South Africa)

Beer production in Zulu society has been investigated by K. Fowler (2006).

His study provides broad indications about the locations of the beer production and the structures associated. It is only mentioned that brewing jars can be found at the back of circular houses and are often partially embedded in the floor (Fowler 2006, 99).

His work includes also accurate data about Zulu ceramic vessels. According to Fowler’s classification, four main types of pottery are used at different stages of the beer manufacturing process (Fowler 2006, 98): (1) Pots used to cook and brew beer: They are very large vessels with a restricted rim and a conical bottom. They come in different sizes and proportions with an average between 44 and 56 cm in height depending on regions (Fowler 2006, 99). These shapes are used for either cooking or fermenting the malt, it is not mentioned whether the same pot may be used for both or if each is specialized. According to the author, smaller shapes are used for daily consumption while large shapes are only used for special occasions. (2) Vessels used to serve beer: They are globular pots with a restricted rim and are distinguished by size and named accordingly. They are on average more than 25, 20, and 15 cm in height depending on the size category (large, medium, and small). The smaller pot can have a ritual function. (3) Pots used to store and serve beer. They are globular shaped pots with a very restricted rim and are wider than they are tall. They range from 30 to 40 cm in height. They are so heavy that smaller vessels are typically used to draw beer until the pot can be lifted. They are only used for special occasions with large gatherings (Fowler 2006, 101). The same shape can be used to store water. (4) Pots used to transport beer. They are used to “store and transport water or beer to work parties in the field or homestead” (Fowler 2006, 102). They are globular with a neck and some vessels classified in a subgroup can have multiple spouts. They vary greatly in size (14-43 cm in height).

### Synthesis

The location of beer-making facilities varies widely from one group to another. Wort boiling and brewing may take place in the compound's courtyard (Dogon, Mouhoun Bend, Mandara), in specialized buildings used exclusively for this function (Mandara), in kitchens that allow this activity (Dogon, Mandara, Gamo, Zulu), or on hearths located outside the compound (Bedik, Mouhoun Bend, Mandara). Beer brewing may take place in any homesteads (Mandara, Bedik) or only in some (Dogon, Gamo). The beer hearths are most often like food cooking hearths, often a little larger (Dogon), but sometimes feature a permanent clay construction incorporating the cooking jars (Mouhoun Bend). When there is a beer hut, it is generally indistinguishable from the other buildings used for other functions, whether in terms of shape and size, or building materials and techniques. Large jars used for fermentation are frequently embedded in the ground to stabilize them across all cultural groups, which induces the presence of wedging pits.

Regarding pottery, all the groups have several types of ceramic vessels used for beer-making, each having very different shapes and sizes. Most of the groups cook the wort and ferment the beer in different jars, except the Gamo who use the same jar for both stages of production. All these containers are large to hold a significant volume of liquid and feature a wide opening. Fermentation jars, with a capacity of 100–200 l, are very similar among Bedik, Dogon, and people of the Mouhoun Bend. Their bottoms are thick and often conical, generated by the roughout technique, or reinforced by the addition of a layer of clay (Dogon, Mandara), to resist thermal shock during the long cooking of beering. Except for the Mouhoun Bend, all groups also have jars for transporting and serving beer, which hold a few l and have very narrow opening. These containers allow fermentation to continue and are even sometimes used as fermentation boosters. Some groups also use medium-sized (15-20 l), narrow-necked jars for storing beer (Mandara), or small containers for storing yeast (Mouhoun Bend). Several also have ritual pots with very special shapes and decorations, on which beer libations are performed (Bedik, Dogon, Mandara). In the only two contexts where use-alteration has been studied (Bedik, Gamo), the vessels in which fermentation took place show characteristic interior attrition. Interior attrition can therefore be considered as an indicator for beer production, but it remains to be clarified if other contents can cause similar alteration, as it seems to be the case with dairy contents (Arthur 2002; 2014).

## Conclusions

The synthesis of the scarce ethnoarchaeological data available on the beer structures (such as beer houses or hearths) and containers (such as pottery) in Africa offers a first overview of the most diagnostic criteria for identifying this fermented beverage in the past societies. In terms of pottery, two main characteristics seem to be highly relevant, regardless the cultural group considered. The first one is the use-alteration visible on the surfaces of the ceramic vessels. Most of the Bedik (Senegal) and Gamo (Ethiopia) pottery involved in the fermentation process of beer show characteristic attrition – or pitting, on their inner surface. The identification of beer-making in archaeology therefore requires the search of this very specific type of use-alteration on the potsherds. Such use-alterations however remains so far scarcely studied due to their difficult reading on the surfaces of pottery due to post-depositional processes and the lack of awareness. The second diagnostic criterion is the shape and size of the ceramic vessels. All the cultural groups (namely Bedik, Dogon, Gamo, Zulu, etc.) use very large jars for germinating grains, cooking, and cooling malt as well as fermenting beer. Most of them also use small to medium-sized ceramic vessels with very narrow openings to ferment, transport and serve beer. The search for such morphological types within the ceramic assemblages seems therefore of a high importance to track beer-making in archaeology. The largest pots tend however to be neglected in pottery studies as they are less likely to show complete profiles compared to the smallest vessels. The development of functional study of pottery, combining typometry and use-alteration, will surely shed new light on the production and consumption of beer in numerous archaeological contexts.

In terms of architecture, only one criterion has proved to be highly relevant, regardless the cultural group considered. These are the shallow pits wedging the large beer pots in the ground. All the cultural groups use them to stabilize the vessels. The identification of beer-making in archaeology therefore requires the search of this very specific type of features. Such shallow pits are however difficult to recognize in archaeology due to the erosion of the sediment layers and the homogeneous filling of the features. Two additional criteria are reliable only in some specific cultural contexts. The first one is the location of the buildings and hearths used to make beer, which is often identical to those used for other activities, except in the Mandara Mountains (Cameroon) where the beer houses have a specific location within the compound, and in the Bedik Country (Senegal) where the beer fireplaces are always located in the outskirts of the compounds. Knowing the distribution of the different structures within an archaeological site however requires large-scale excavations, which are rarely carried out. The second criterion is the shape and the size of the beer houses and hearths, as well as their building materials and techniques. If they are most often like the structures used for other activities, there are places like in Burkina Faso where the clay structures used to cook malt are very different from other fireplaces. However, there are no evidence so far of such earthen ovens preserved in archaeological sites. In the current state of research, the architectural analysis of structures does not seem a promising approach to highlight beer-making in past societies.

Too few ethnoarchaeological studies focused on the identification of beer-making in Africa have been carried out to date. In the future, it would be necessary to extend the surveys to other present-day cultural groups to better grasp the constants and variations in terms of beer structures and containers and refine the criteria for identifying this fermented beverage. At the same time, it would be useful to increase archaeological research to track the production and consumption of beer in the past societies. With this strategy, combined with organic residue analysis in search for biomarkers of fermentation (ongoing research; Drieu et al. 2022), it should be possible to trace back the history of this fermented beverage in Africa over several centuries or millennia, and gain a better understanding of its evolution, in terms of ingredients, technical processes and gestures, organization of production and related socio-economic and religious implications.

## Acknowledgements

This ethnoarchaeological study has benefited from an authorization for archaeological surveys and excavations in this region, signed in 2021 by the Director of the Cultural Heritage of Senegal. Local permission to excavate in Eguong was given by the direct descendants of the former inhabitants of the abandoned compound, currently living in Andiel and Mangama, as well as the inhabitants of Iwol, the closest village.

We would like to thank the Swiss National Science Foundation for its generous grant, the administrative persons in the ARCAN laboratory at the University of Geneva for their much-appreciated support, our colleagues at UCAD and MCN in Dakar, as well as all the colleagues and students of the different teams of the Sinergia project for their enthusiastic collaboration. We would also like to thank warmly all the Bedik people with whom we worked and tasted food and beer for their welcoming, hospitality and the sharing of their deep knowledge.

Finally, we would like to thank the reviewers and editors for their dedication to the improvement of this paper, through fruitful comments and discussions. Their intervention has made a significant impact on this paper.

## Supplementary Material

Supplemental Material
