# Supplementary material for: Identifying Past Beer Production: Contributions from an Ethnoarchaeological Study in Bedik Villages, Senegal
Source: Ethnoarchaeology. 2024 Apr 16;16(1):126–62. doi: 10.1080/19442890.2024.2334509 (PMC11184625; doi:10.1080/19442890.2024.2334509)
Supplement: Supplemental Material [file YETH_A_2334509_SM6262.zip › Appendix 2.docx]

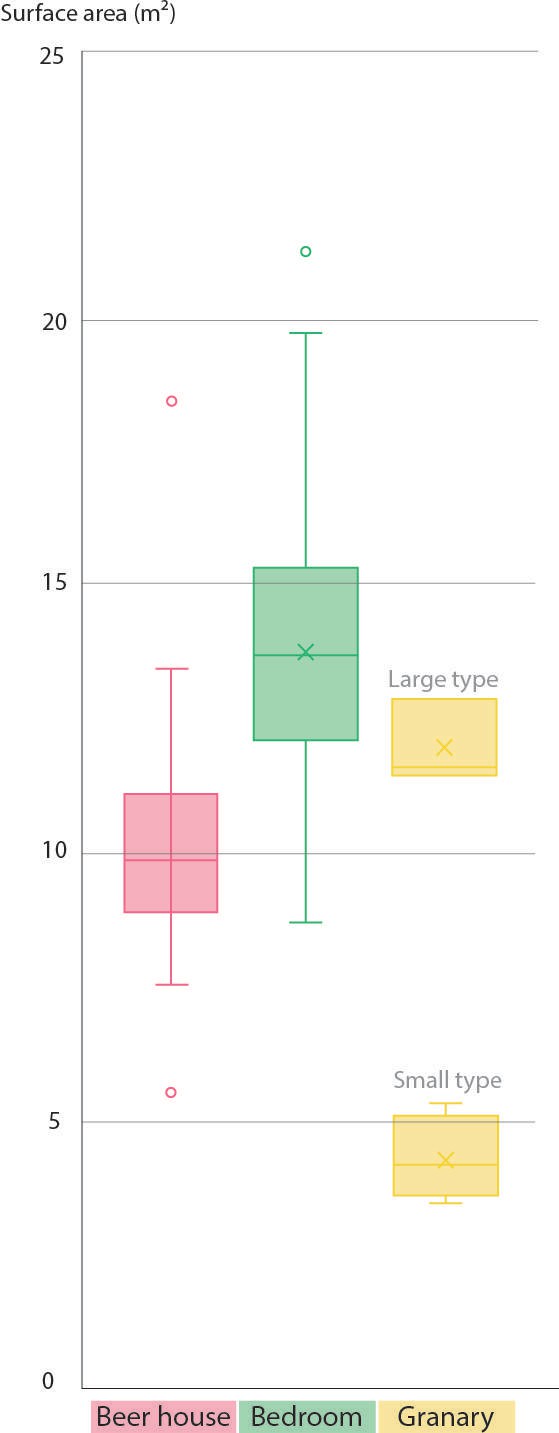
Supplement 2: Surface area of beer houses (left), bedrooms (center) and granaries (right) in present-day Bedik villages (Iwol, Ethwar, Andiel, Manda Thiès, Ninefesha, Indar).
