# Supplementary material for: Identifying Past Beer Production: Contributions from an Ethnoarchaeological Study in Bedik Villages, Senegal
Source: Ethnoarchaeology. 2024 Apr 16;16(1):126–62. doi: 10.1080/19442890.2024.2334509 (PMC11184625; doi:10.1080/19442890.2024.2334509)

Supplement 3. Inner layouts of present-day beer houses: (a) shallow pit used to maintain large ceramic vessels; (b) Altar in an abandoned beer house (Andiel).


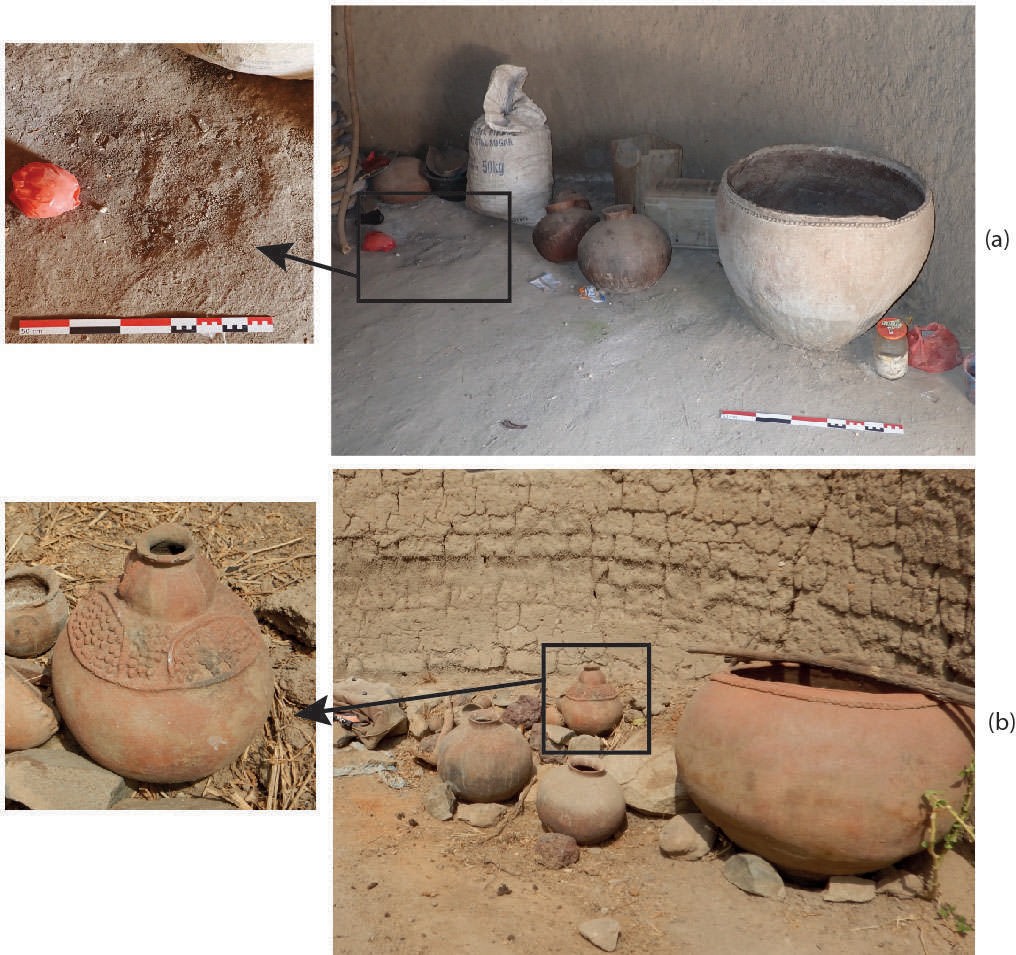

Supplement: Supplemental Material [file YETH_A_2334509_SM6262.zip › Appendix 3.docx]
