# Supplementary material for: Identifying Past Beer Production: Contributions from an Ethnoarchaeological Study in Bedik Villages, Senegal
Source: Ethnoarchaeology. 2024 Apr 16;16(1):126–62. doi: 10.1080/19442890.2024.2334509 (PMC11184625; doi:10.1080/19442890.2024.2334509)
Supplement: Supplemental Material [file YETH_A_2334509_SM6262.zip › Appendix 4.docx]

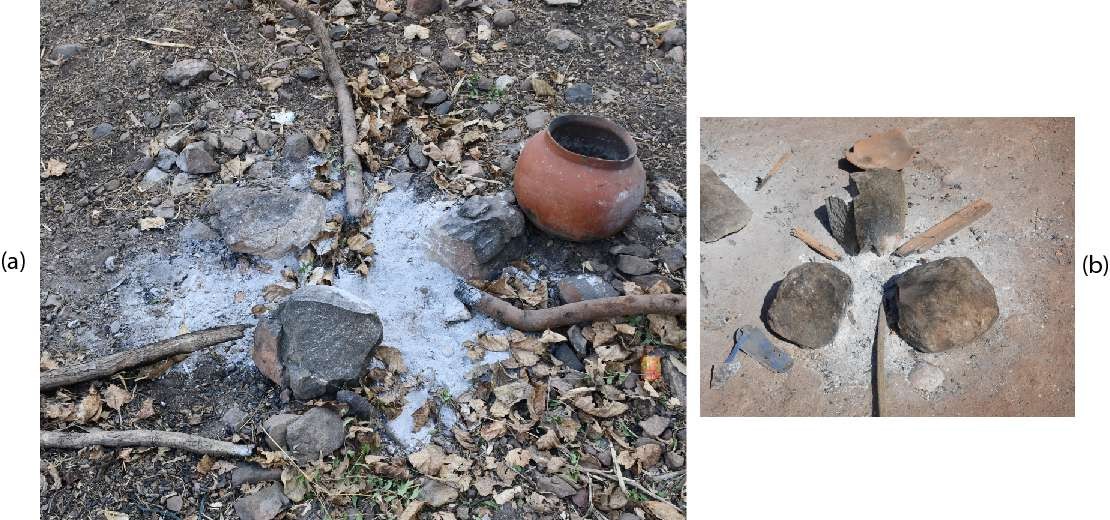
Supplement 4. Examples of cooking areas in Bedik present-day villages: (a) hearth used for beer (Andiel); (b) hearth used to cook meals (Andiel).
