# Supplementary material for: Identifying Past Beer Production: Contributions from an Ethnoarchaeological Study in Bedik Villages, Senegal
Source: Ethnoarchaeology. 2024 Apr 16;16(1):126–62. doi: 10.1080/19442890.2024.2334509 (PMC11184625; doi:10.1080/19442890.2024.2334509)

Supplement 5: Surface area of the beer house (left), bedrooms (center) and granaries (right) identified in the abandoned compound of Eguong.


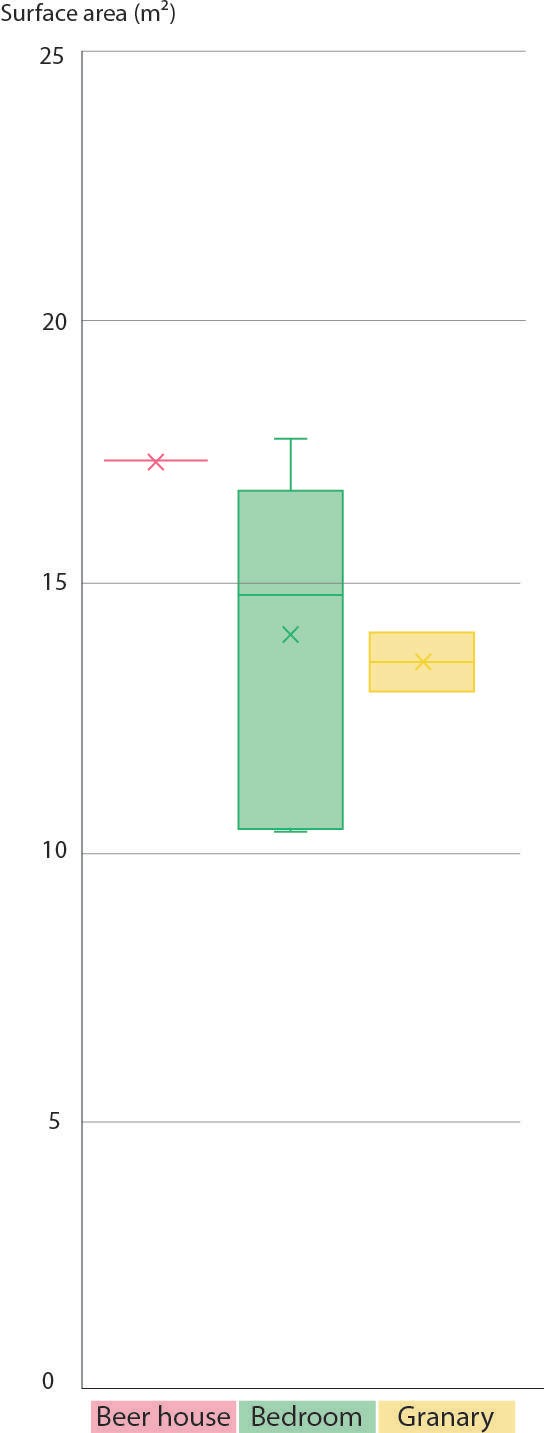

Supplement: Supplemental Material [file YETH_A_2334509_SM6262.zip › Appendix 5.docx]
